# Supplementary material for: Electrochemical-repaired porous graphene membranes for precise ion-ion separation
Source: Nat Commun. 2024 May 13;15:4006. doi: 10.1038/s41467-024-48419-6 (PMC11091207; doi:10.1038/s41467-024-48419-6)
Supplement: Supplementary file 1 — Supplementary Information [file 41467_2024_48419_MOESM1_ESM.pdf]

Supplementary Information

Electrochemical-repaired porous graphene membranes for precise ion-ion separation

Zongyao Zhou[1,2], Kangning Zhao[1], Heng-Yu Chi[1], Yueqing Shen[1], Shuqing Song[1], Kuang-Jung Hsu[1], Mojtaba Chevalier[1], Wenxiong Shi[3], Kumar Varoon Agrawal[1*]

[1] Laboratory of Advanced Separations (LAS), École Polytechnique Fédérale de Lausanne (EPFL), Sion, CH-1950 Switzerland

[2] School of Environment, Harbin Institute of Technology, Harbin 150090, P. R. China

[3] Institute for New Energy Materials and Low Carbon Technologies, School of Materials Science and Engineering, Tianjin University of Technology, Tianjin 300387, P. R. China

[*]Email: kumar.agrawal@epfl.ch

Table of Contents

Supplementary Notes

Supplementary Note 1:
Preparation of CNT water solution.

Supplementary Note 2:
Preparation and characterizations of CNT free-standing films.

Supplementary Note 3:
Surface structures and morphologies of the CNT-graphene membranes.

Supplementary Note 4:
Mechanical stability of the CNT-graphene membranes.

Supplementary Note 5:
Pore size quantification.

Supplementary Note 6:
Electropolymerization of CMP mask layer.

Supplementary Note 7:
Surface structures and morphologies of the CMP-masked graphene membranes.

Supplementary Note 8:
XPS depth-profiling analysis.

Supplementary Note 9:
Membrane structure analysis.

Supplementary Note 10:
Raman spectrum of the CMP-masked Gr membrane.

Supplementary Note 11:
Zeta potential analysis.

Supplementary Note 12:
Simulation analysis of the interaction between CMP and graphene.

Supplementary Note 13:
Ion sieving performance of the CMP films.

Supplementary Note 14:
Effects of the CMP thickness on the ion sieving performance.

Supplementary Note 15:
Effects of concentration on ion sieving performance.

Supplementary Note 16:
Ion sieving performance under electric field-driven process.

Supplementary Figures

Supplementary Figure 1. Optical photographs of a) the CNT solution, b) the vacuum filtration setup, and c) CNT/PES membrane.
Supplementary Figure 2. Morphology of the CNT/PES support. a) Surface SEM image of the PES support, b), c) and d) surface SEM images of the CNT/PES support.
Supplementary Figure 3. C1s XPS spectra from the free-standing CNT film.
Supplementary Figure 4. Raman spectra of the free-standing CNT film.
Supplementary Figure 5. Morphology of the CNT free-standing film. a) Surface SEM image of the CNT free-standing film, b) cross-sectional SEM image of the CNT free-standing film.
Supplementary Figure 6. Morphology of the free-standing CNT film. a) TEM image of the CNT free-standing film, b) a SAED pattern from the region shown in c) of the free-standing CNT film.
Supplementary Figure 7. Optical photographs of a) the free-standing CNT film floating on DI water, and b) the CNT/graphene/Cu.
Supplementary Figure 8 Morphology of the CNT-supported monolayer graphene under a) detector T2, b) detector T1, and c) detector ETD.
Supplementary Figure 9. Morphology of the CNT-supported monolayer graphene under a) detector T2, b) detector T1, and c) detector ETD.
Supplementary Figure 10. TEM image of the CNT-supported graphene.
Supplementary Figure 11. Mechanical stability of a CNT-Graphene membrane.
Supplementary Figure 12. Water flux of the membrane before and after the abrasion test.
Supplementary Figure 13. SEM images of the membrane before and after the abrasion test.
Supplementary Figure 14. Water permeance of the CNT support and the CNT-supported pristine graphene.
Supplementary Figure 15. Schematic diagram of the membrane module used in the pressure-driven nanofiltration performance tests.
Supplementary Figure 16. Optical photographs of a) the membrane module and b) the sealed membrane, c) and d) optical photographs of the setup used in the pressure-driven nanofiltration performance tests.
Supplementary Figure 17. C1s XPS spectra of a) pristine graphene, b) O_3-treated graphene, and c) 5 min of CO_2-treated graphene.
Supplementary Figure 18. Raman spectra of a) pristine graphene, b) O_3-treated graphene, and c) 5 min of CO_2-treated graphene.
Supplementary Figure 19. Schematic of the measurement of the pore size in the graphene.
Supplementary Figure 20. Schematic diagram of the setup used in the concentration-driven performance tests.
Supplementary Figure 21. Schematic of the electropolymerization device.
Supplementary Figure 22. Mechanism of electropolymerization of CMP mask layer.
Supplementary Figure 23. Current curves recorded in the first CV cycle.
Supplementary Figure 24. AFM images of the CMP films with a) 30 CV cycles, b) 40 CV cycles, c) 50 CV cycles, and d) membrane thickness *vs.* number of CV scans.
Supplementary Figure 25. Surface SEM images of the top and back sides of the membranes.

Supplementary Figure 26. AFM image of the 5cCMP-Gr membrane.
Supplementary Figure 27. Cross-section TEM images of CMP-masked Gr membranes.
Supplementary Figure 28. XPS depth profile analysis.
Supplementary Figure 29. a) Schematic structures of the membrane, b) schematic of CMP polymer covered CNT, and c) TEM image of CMP polymer covered CNT.
Supplementary Figure 30. Raman spectrums of CMP-masked Gr and CNT support.
Supplementary Figure 31. Zeta potential of a) CNT support, b) porous Gr on CNT support, c) CMP on CNT support and d) CMP-masked Gr on CNT support.
Supplementary Figure 32. Simulated structures of CMP-masked Gr membrane.
Supplementary Figure 33. Simulated structures and pore size distribution.
Supplementary Figure 34. Diffusion comparison of Li^+ through porous graphene membranes with and without CMP mask layer.
Supplementary Figure 35. Ion sieving performance of the CMP-CNT (without graphene) prepared under different scan rates (50 mV/s, 200 mV/s, and 300 mV/s) with 5 CV cycles.
Supplementary Figure 36. SEM images of the membrane 5c50CMP on CNT support (without graphene) under different magnification.
Supplementary Figure 37. Ion sieving performance of the CMP-masked graphene (treated by 6-second plasma) membranes.
Supplementary Figure 38. Ion sieving performance of the CMP-masked graphene (treated by 5-minute CO_2) membranes with various CMP layers prepared with 3, 5, and 10 CV cycles under the scan rate of 200 mV/s.
Supplementary Figure 39. Ion sieving performance as a function of pH.
Supplementary Figure 40. a) Ion permeation rate as a function of salt solution concentration, b) ion permeation rate with unit normalized by the concentration difference.
Supplementary Figure 41. Long-term binary ion sieving performance of the CMP-masked graphene (treated by 5-minute CO_2) membranes.
Supplementary Figure 42. Long-term a) K^+/Mg^{2+} and b) Na^+/Ca^{2+} binary ion sieving performance of the CMP-masked porous graphene membrane.
Supplementary Figure 43. Trade-off relationship between K^+/Mg^{2+}selectivity and K^+ permeation rate of the CMP-masked graphene membranes and the state-of-the-art ion-sieving membranes reported in the literature, tested under concentration-driven single-ion process.
Supplementary Figure 44. Schematic of the setup for the ion sieving tests under electric field.
Supplementary Figure 45. Current-voltage (*I*-*V*) characteristics CMP-masked Gr membranes recorded in LiCl, $CaCl_2$, and $MgCl_2$ solution.

Supplementary Tables

Supplementary Table 1. Selected physical and thermodynamic characteristics of the tested ions.

Supplementary Table 2. Comparison of the membranes for Li^{+}/Mg^{2+} separation in concentration-driven single-ion sieving tests.

Supplementary Table 3. Comparison of the membranes for K^{+}/Mg^{2+} separation in concentration-driven single-ion sieving tests.

Supplementary References

Supplementary Note 1

Preparation of CNT water solution

CNT solution was prepared by a sample vacuum filtration method as reported before[1,2]. Initially, 0.1g CNT powder (purity>95%, length 5−30 μm, XFnano), and 1g sodium dodecylbenzenesulfonate were added into 1 L deionized (DI) water. The mixture was sonicated using a probe ultrasonicator at 35 W. Subsequently, the obtained mixture solution was centrifuged at 10,000 rpm for 30 minutes to remove any undispersed CNT powder. After centrifugation, 800 mL of the supernatant solution was carefully collected. Then, 50 mg of dopamine hydrochloride and 80 mL of HCl-tris buffer (0.1 mol/L, pH=8.5) were added to the solution. The resulting solution was stirred at 40°C for 6 hours to ensure thorough mixing and reaction. Finally, the CNT water solution was obtained after undergoing two additional centrifugation steps at 6000 rpm for 30 minutes each. The prepared CNT water solution was diluted three times before use and stored at room temperature (Supplementary Figure 1a).

Supplementary Note 2

Preparation and characterizations of CNT free-standing films

First, 20 mL CNT water solution was filtrated on a commercial hydrophilic polyethersulfone (PES) substrate to obtain a CNT-PES membrane via a vacuum filtration setup as shown in Supplementary Figure 1b. The resulting CNT-PES membranes (Supplementary Figure 1c) then were dried in an oven at 70 °C. The CNT free-standing film was fabricated by immersing the pre-prepared CNT-PES in a dimethylformamide (DMAc) solution[1]. Once the PES support had been dissolved, the CNT free-standing film was transferred into DI water using a nylon mesh to eliminate any remaining DMAc solution. To ensure the complete removal of any residual PES polymer residue and obtain a pure CNT free-standing film, the etching process was repeated at least three times, involving the sequential transfer of the CNT film between DMAc and DI water.

Surface SEM images (Supplementary Figure 2) demonstrate that the CNT-PES membrane exhibits a consistent and porous CNT network structure that envelops the underlying PES substrate. As the thickness of the CNT layer increases, the SEM images no longer reveal the presence of large pores on the PES substrate beneath the CNT network. This can be conveniently regulated by adjusting the volume of the CNT solution during the fabrication process. The XPS spectra (Supplementary Figure 3) show that the surface of the CNT film contains oxygen, nitrogen, and carbon functional groups[2,3]. Deconvolution of the C1s peak unveils that the carbon signals come from both CNT and polydopamine, but mainly from the sp^2 carbons of CNT. The characteristic C peak at 288.8 eV[1-3] observed in this spectrum indicates the successful modification of polydopamine (PDA) to the CNT. The Raman spectra (Supplementary Figure 4) exhibit three peaks, which are well consistent with the reported D band, G band, and 2D band of the CNT carbon structure[3].

The thickness of the optimized CNT film was determined to be an average of 270 nm, as indicated in the cross-sectional SEM image (Supplementary Figure 5) and cross-sectional TEM image (Supplementary Figure 27). The TEM results reveal that the CNT network exhibits an interlocked array structure of nanotubes (Supplementary Figure 6a). The SAED pattern displayed well-ordered diffraction rings (Supplementary Figure 6b), indicating the polycrystalline nature of the CNT film. However, a distinct graphene characteristic diffraction pattern was clearly observed following the transfer of a single-layer graphene onto the CNT support as discussed in the main text (Figure 1c). It suggests the successful transfer of graphene from copper substrate to the CNT support and the good structural integrity of the graphene.

Supplementary Note 3

Surface structures and morphologies of the CNT-graphene membranes

The structures and morphologies of the CNT-supported membranes were checked by SEM. It should be noted that no metal coating was used in the SEM experiments. Normally, observing a single-layer graphene with a standard scanning electron microscope (SEM) can be challenging due to the low interaction between high-energy electrons used in SEM and the single-layer graphene sheet. The low interaction results in weak contrast, making it difficult to distinguish the graphene layer from the substrate and obtain clear images. As shown in Supplementary Figures 8b, 8c, 9b, and 9c, the demarcation between the graphene and CNT support is challenging to distinguish in such cases because the thickness of a single-layer graphene should be only 0.34 nm[4].

To observe the edge of graphene and CNT support in SEM, a backscattered electron detector was utilized. The detector is used to gather compositional and material contrast information, while a secondary electron detector is used for surface morphology and topography. Backscattered electrons are higher in energy compared to secondary electrons and can provide different compositional and material contrast information. Although CNT has the same composition as graphene, the CNT in this work is modified by polydopamine. The difference in composition and materials between the support and graphene makes the single-layer graphene more visible. The SEM images obtained using the backscattered electron detector (Figure 1b, Supplementary Figures 8a, and 9a) showed that a fully continuous graphene layer was well supported by an underlying uniform and porous CNT network. Supplementary Figure 9a showed a structurally intact graphene with a larger size than that in Supplementary Figure 8a. The graphene layer is tightly attached to the porous CNT support and the underlying CNT support is clearly discernible in the image.

The clear observation of the graphene layer on the CNT support under SEM strongly indicates the successful transfer process and the structural integrity of the graphene. The graphene supported by CNT was also checked by TEM (Supplementary Figure 10). The graphene characteristic diffraction pattern (Figure1c) further supports the successful transfer of the graphene.

Supplementary Note 4

Mechanical stability of the CNT-graphene membranes

It should be noted that in our experiments, there were no instances of the graphene layer peeling off from the CNT film, which suggests a robust affinity between the CNT and graphene. This strong bonding is likely attributed to the following main factors [5]: i) robust van der Waals forces and ii) π-π interactions between the CNT and graphene. Additionally, the introduction of polydopamine (PDA) as a binder[6] played a role in establishing an architectural bridge between the CNT and graphene, further enhancing the interfacial adhesion. Furthermore, owing to the exceptionally high specific surface area of the CNT-graphene membrane, it exhibits strong adherence to the PTFE support, particularly following a drying treatment.

The mechanical stability of the CNT-graphene membrane is evaluated by its resistance to external abrasion[5]. As shown in Supplementary Figure 11, a P2000 sandpaper, with a weight of 6.4 g applied as pressure, was employed to scrub the membrane. Remarkably, the membrane displayed no visible signs of alteration or damage throughout the test. Following the abrasion experiment, the membrane (before pore etching) retained nearly identical water flux values (~0.3 $L/m^2/h/bar$ on average, Supplementary Figure 12), suggesting the structural integrity of the graphene. Due to the orientation of the graphene layer facing the PTFE support, the PTFE-graphene-CNT sandwich structure imparts excellent resistance to external abrasion and water flushing to the graphene layer. Moreover, although the CNT side comes into direct contact with the sandpaper, its surface morphology remains unchanged, as confirmed by SEM analysis, as depicted in Supplementary Figure 13. All the results indicate the good mechanical stability of the CNT-graphene membrane.

Supplementary Note 5

Pore size quantification

The pore diameter is determined through the analysis of TEM images. After the image processing, a sphere fitting method is employed to determine the effective pore diameter. Specifically, a circle is fitted inside a pore by locating the outermost carbon atoms at the edge. The resulting diameter (D_I) is obtained as illustrated in Supplementary Figure 19. This circle contacts the outermost edge of the carbon atoms. Subsequently, adding the atomic radii of carbon atoms, we constructed another circle intersecting the center of these edge carbon atoms. The diameter of this circle is denoted as D_C. Next, we subtracted the nonbonded interaction length between graphitic carbon and a water molecule, represented by 2^ (1/6) σ (=3.86 Å) corresponding to the position of the potential well[7], from D_C. This subtraction yields an effective pore diameter.

Supplementary Note 6

Electropolymerization of CMP mask layer

In this study, 1,3,5-tris(N-carbazolyl) benzene (TCB) served as the building block for CMP. Molecular simulations[8] indicated its capability to form a CMP polymer with a pore size from 0.85 to 1 nm, surpassing D_H of Mg^{2+} ion. Consequently, the CMP layer is not anticipated to exhibit selectivity for mono/divalent ions. Both the experimental findings in this study and that in the literature[8] corroborate that the CMP layer demonstrates negligible Li^+/Mg^{2+} selectivity (further discussion in Supplementary Notes 13 and 14), aligning with the simulation predictions.

The polycarbazole CMP mask layer was synthesized through continuous cyclic voltammetry (CV) in the voltage range of -0.80 V to 1.03 V (*vs*. Ag/Ag^+). The CV curve is composed of both a positive and a negative segment. In the positive CV scan, the carbazole units of TCB underwent oxidation[8,9], with the onset oxidative potential observed at around 0.9 V. The resulting carbazole radicals subsequently coupled to form dimeric carbazole cations, as illustrated in Supplementary Figure 22. A reductive peak appeared at 0.64 V in the negative scan (Supplementary Figure 23), indicative of the reduction of the dimeric carbazole cations. From the second scan onward, a new and stable oxidative peak emerged at 0.75 V, corresponding to the oxidation of the formed dimeric carbazoles[9]. Subsequent cathodic sweep scans reduced the cation radicals back to their neutral state. Further reactions extended the polymer chain and reduced solubility. Ultimately, the CMP film was deposited in situ on porous graphene. The film thickness could be precisely controlled by adjusting the number of CV scans[9]. The membrane thickness increased linearly corresponding to the number of CV scans, exhibiting a growth rate of ~ 2 nm per CV cycle as discussed in the main text (Supplementary Figure 24 and Figure 3d). Notably, achieving such precise control over the thickness of a polymer film is challenging using traditional synthetic methods.

Supplementary Note 7

Surface structures and morphologies of the CMP-masked graphene membranes

Supplementary Figures 25b, 25b1, and 26 show the morphologies of the CMP-masked porous graphene membrane. The CMP layer was prepared under 5 CV cycles (5cCMP-Gr-CNT). The CNT side of the 5cCMP-Gr-CNT membrane has very similar structures to that of the membrane without CMP (Supplementary Figure 25a). It should be noted that the CMP polymer can grow from both sides of the Gr-CNT membrane when the membrane is exposed to the CMP monomer solution[3]. The CMP monomers could diffuse within the CNT network film and disperse around the graphene layer. Given the electric conductivity of both graphene and CNT, the CMP polymer forms wherever monomers are present under the applied voltage, and deposits as film on the surfaces of both graphene and CNT. However, due to the larger space available inside of the CNT film, no noticeable CMP polymer film can be observed on its surface with 5 CV cycles of growth of the CMP. In other words, CMP polymer grew inside the CNT network[3,8]. In contrast, it is easier to get a continuous CMP layer on the graphene surface. The minute granular structures were observed on the graphene side, suggesting the CMP layer. With an increase in CV cycles to 30, the thickness of the CMP layer becomes larger, and the granular structures on its surface become increasingly clear on the graphene side (Supplementary Figure 25c). Additionally, the CMP polymer is obvious on the CNT side (Supplementary Figure 25c1). The gaps between the CNT are partially filled by the CMP on the CNT side, but the CNT network is still visible. It's intriguing to highlight that the contrasting morphologies observed in the CMP layer on each side of the CNT-Gr membrane further substantiate the presence of the graphene on the CNT support.

Supplementary Note 8

XPS depth-profiling analysis

To accurately detect the components near the graphene layer, we conducted a pre-experiment to determine the etching thickness via controlling the etching parameters (sputter time, accelerating voltage, current, and so on). A freestanding porous graphene was transferred to a silicon wafer coated with 100 nm thick Au metal. After the electropolymerization, the sample CMP-porous Gr-Au-Si was obtained. The graphene layer is attached to the Au metal layer, the etching conditions are determined once the Au element is detected. Next, we employed the same etching conditions, ensuring the detection of elements near the graphene layer, to etch the CMP-masked Gr membranes with a CNT support (CMP-porous Gr-CNT-PTFE). Finally, the sputter time was set to 0s, 50s, and 100s to detect the components on the surface of the sample, near the porous graphene layer, and the CNT support, respectively. After each etching, the XPS spectra of C and N were recorded for comparison.

The evolution of C1s and N1s peaks as a function of sputter time for the samples is shown in Supplementary Figure 28. After fitting, the C1s spectrum obtained from the sample surface (sputter time=0s) could be split into two peaks attributed to C-C/C-H/C=C at 284.8 eV and C-N/C-OH at 286.7 eV of the CMP polymer[10]. After sputtering for 50 seconds, the C1s spectrum showed no significant changes, suggesting that the CMP polymer near the porous graphene layer is almost the same as the CMP on the surface. Further increasing the sputtering time to 100 seconds, a new peak at 289.0 eV was observed in the C1s spectrum, which should be attributed to N-C=O/O-C=O from the polydopamine covered on CNT[2,11].

The N1s spectrums as a function of sputter time are shown in Supplementary Figure 28d. The N1s spectrum obtained from the surface CMP (sputter time=0s) shows one main peak, which should be attributed to the R_2-NH group in the CMP polymer[12]. The characteristic R-NH_2 peak of dopamine at 401.4 eV is not observed in this spectrum. It indicates that a CMP layer uniformly covers the porous graphene-CNT layer. After etching for 50 seconds, the R-NH_2 peak was observed, which is associated with dopamine and polydopamine[11]. The N1s spectrum obtained at 50 s is similar to that obtained with a sputtering time of 100 seconds. The latter reaches the depth of the CNT support layer. Although etching for 50 seconds should reach the depth of the graphene layer, it still collects signals from the underlying CNT layer. It is reasonable because the XPS detection signal originates from a depth of several nanometers from the sample surface, especially, given that graphene is only 0.34 nm in thickness. The XPS depth-profiling analysis helps confirm the multilayer structure of the membrane.

Supplementary Note 9

Membrane structure analysis

After a comprehensive set of characterizations, including XPS depth-profiling, TEM FIB cross-section, TEM, and SEM, we conclude that the membrane's structure is likely as follows (Supplementary Figure 29):

The CMP monomers will distribute on the surface and inside the CNT support of the membrane once the Gr-CNT membrane is in contact with the reaction solution. Next, under the action of an electric field, the CMP monomers become CMP polymer after a series of oxidation and reduction reactions. The reactions extended the polymer chain and reduced its solubility. Ultimately, a CMP film was deposited *in situ* on the conductive substrate. Since both graphene and CNT possess excellent conductivity, CMP grows and deposits on the surfaces of both Gr and CNT as shown by the following schematic. It is easier to form a continuous thin film by depositing it on the surface of the graphene layer (top side of the membrane). Therefore, it is easy to observe a CMP polymer layer on this side under SEM. However, it needs more polymer to form a continuous film on the CNT side (bottom side), because of the relatively large space and specific surface area inside the CNT network support. On the CNT side, the polymer first covered the CNT bundles filled all the gaps between the CNT, and then formed a continuous thin film. Therefore, the morphologies of the two sides are very different, which is supported by the SEM results (Supplementary Figure 25). Additionally, the structure of the membrane is also supported by TEM FIB and XPS results.

Supplementary Note 10

Raman spectrum of the CMP-masked Gr membrane

The Raman spectrum of the CMP-masked Gr membrane showed typical characteristics of carbon nanotubes with a D-band (1363 cm^{-1}), a G-band (1593 cm^{-1}), and a 2D-band (2717 cm^{-1}). Considering that the XPS depth profile analysis, SEM surface images, and TEM cross-section images all confirm the presence of a continuous CMP mask layer on top of porous graphene, we attribute this phenomenon to the influence of the underlying CNT support. To further investigate this hypothesis, we fabricated a thicker CMP polymer film with a thickness of ~20 nm and checked its Raman spectra. The result (Supplementary Figure 30) obtained from the thicker membrane consistently aligns with that obtained from the CNT support, affirming our hypothesis.

Supplementary Note 11

Zeta potential analysis

Although there are positively charged amines in Supplementary Figure 22. They are carbazole cations, which are the intermediate products of oxidation half-reactions. These positively charged cations undergo a reduction in the following reduction half-reactions. Additionally, the reductive peak appeared at 0.64 V in the negative scan, but the reductive current was set as low as -0.8 V, ensuring that the product was completely reduced to a neutral state. Therefore, from a chemical perspective, the CMP polymer cannot carry positive charges.

Regarding the charge properties, the zeta potential of the CNT support, porous Gr, CMP membrane, and CMP-masked Gr have been checked and compared. All samples showed negative surface charge from pH=3 to pH=10, which is due to strong negative charge on CNT. It should be noted that the CMP film itself is not charged, which was investigated and reported in the literature[9]. The porous graphene prepared by CO_2 expansion at 800 ºC is devoid of functional groups. Indeed, functional groups are not detected by XPS as shown in Supplementary Figure 17c. The reason for these membranes to be negatively charged would be due to the influence of the negatively charged polydopamine CNT substrate. Even though the porous CMP polymer has a thickness of ~10 nm, the effects of this substrate on the upper layer's potential have been widely reported[13,14].

Supplementary Note 12

Simulation analysis of the interaction between CMP and graphene

In the main text, simulations are utilized to explore the pore size distribution (PSD) at the interface of the CMP-masked Gr membrane. These simulations are essential as because masked graphene pores cannot be visualized in TEM. Herein, the focus is on investigating the interaction between CMP and porous graphene, where pores in graphene would represent selective membrane (e.g., small pores generated by $5CO_2$-Gr) and nonselective membrane (e.g., large pores generated by 6s plasma-Gr).

For selective membrane, eight small (selective) and large (non-selective) pores were generated. This represented sample obtained after a 5-minute CO_2 treatment (5min CO_2-Gr). For nonselective membrane, four large pores were generated with sizes close to 2.4 nm. This represented graphene treated with plasma for 6 seconds (6s plasma-Gr) where pores are approximately ~2.4 nm on average, as reported in our previous work [13].

Next, we conducted simulations for 5min CO_2-Gr. The atomic position in the film was frozen to keep all the positions fixed. Upon achieving system stability, the 5min CO_2-Gr graphene was replaced by the 6s plasma-Gr graphene, keeping the CMP film same. The structure of 5min CO_2-Gr and 6s plasma-Gr are different, but the lattice positions were the same. Therefore, the "CMP masked 5min CO_2-Gr" and "CMP masked 6s plasma-Gr" systems are almost the same, except for the pores on the graphene. Then Zeo++ was employed to analyze the PSD of the membranes after being masked by the same CMP film.

It is essential to highlight that, for a more comprehensive exploration of the interaction between the CMP polymer and porous graphene, a distinct strategy was used in these simulations compared to that detailed in the main text. Instead of measuring the PSD of the entire membrane, comprising a 6-nm-thick CMP layer and a porous graphene layer, the focus was on investigating the interface between the CMP layer and the graphene layer. Specifically, a 6-nm-thick CMP layer was designed atop the porous graphene, and the CMP polymer was "sliced" at a distance of 1 nm from the graphene layer with the slicing direction parallel to the plane of graphene. Subsequently, a statistical analysis of the PSD of the porous graphene (encompassing both $5CO_2$-Gr and 6s plasma-Gr) with the 1-nm-thick CMP polymer, closest to the graphene layer, was conducted.

The results depicted in Supplementary Figures 33a-d reveal that in the CMP-masked 5minCO_2-Gr membrane, denoted as "CMP-masked-Gr" in the main text, more than 95% of the pores possess a size smaller than 0.86 nm (corresponding to the hydrated diameter of Mg^{2+}). This implies a significant limitation on the diffusion of divalent ions. Simultaneously, no pores exceeding 0.9 nm are recorded, suggesting that all large pores are effectively masked by the CMP layer and segmented into smaller ones. The defects in the porous graphene have been successfully repaired. As discussed in the main text, CMP polymer effectively partitions non-selective large pores into distinct smaller ones, thereby narrowing the PSD. This observation is reminiscent of the pore-in-pore strategy, wherein the interplay between the pores in the graphene and those in the CMP leads to an overall reduction in pore size. However, in comparison, for the 6s plasma-Gr sample (Supplementary Figures 33e-h), upon being masked by the 1-nm-thick CMP layer, the predominant feature in the PSD curve is the existence of pores surpassing 0.8 nm, although the larger pores exceeding 2 nm disappeared. Approximately 50% of the membrane pores lie within the range of 0.7 nm to 1.0 nm. Significantly, this aligns with the PSD observed in the CMP polymer itself, as detailed in the literature[2]. This occurs due to the diminishing synergistic effect between the large pores (exceeding 2 nm) in the graphene and those in the CMP polymer, leading to increased exposure of the pores inherent to the CMP polymer. Notably, these CMP pores are larger than the hydration diameter of Mg^{2+}. Consequently, in ion tests, the CMP-masked 6s plasma-Gr membrane exhibits no discernible Li^+/Mg^{2+} selectivity. To a certain extent, the CMP polymer is limited in its ability to further reduce the pores in the 6s plasma graphene, as this is dictated by the intrinsic pore size of the

CMP material.

The simulation experiments conducted above underscore the importance of precisely controlling the pore size in graphene membranes. Although CMP effectively narrows the pores in porous graphene, the ion selectivity is derived from the synergistic interaction between CMP and graphene pores. In this investigation, the micropores within the CMP polymer itself demonstrate inefficacy in achieving selectivity between monovalent and divalent ions. Even in the case of the CMP-masked 6s plasma-treated graphene, efficient ion separation remains elusive, primarily attributed to the excessively large size of the graphene pores.

Supplementary Note 13

Ion sieving performance of the CMP films

The ion sieving performance of the CMP films (without the porous graphene layer) prepared under various electropolymerization conditions was checked (Supplementary Figure 35). The membrane 5c200CMP was prepared directly on a CNT support with 5 CV cycles and a scan rate of 200 mV/s, using the same conditions for the preparation of CMP-masked Gr membranes. In addition to the preparation of 5c200CMP, 5c50CMP, and 5c300CMP membranes were also prepared as controlled samples under a scan rate of 50 mV/s and 300 mV/s, respectively. The ion sieving results show that the Li^{+}/Mg^{2+} selectivity of these CMP membranes without the porous graphene is negligible. This is also corroborated by prior literature on CMP[8]. The highest selectivity is lower than 2. It should be noted that 5c50CMP-CNT (50 mV/s, 5 CV cycles) features a continuous CMP layer on the CNT network, fully filling the gaps between CNT bundles, as confirmed by the SEM images (Supplementary Figure 36). The membrane's surface exhibits typical granular structures, with the underlying CNT being invisible. Although the membranes 5c50CMP-CNT have a continuous structure, the Li^{+}/Mg^{2+} selectivity is still negligible with a value of 1.2. It indicates that the porous graphene layer plays the role of the selective layer in the membranes, and the CMP layer itself is nonselective to the Li^{+}/Mg^{2+}. This result further confirms the synergistic effects between the CMP and the porous graphene on the ion selectivity. As discussed in the main text, the CMP mask layer can block, partition, and convert the non-selective large pores in the graphene into selective smaller ones, providing an enhanced uniformity to the pore size distribution (PSD) of the membrane. As a result, the transport rate of the divalent ions is inhibited due to their relatively larger hydration size compared with that of the monovalent ions, thus enhancing the Li^{+}/Mg^{2+} selectivity. The results also indicate that the scan rate utilized in the electropolymerization process has discernible impacts on the thickness and/or pore size of the CMP layer (more specific investigations are needed as a distinct project). Notably, the 5c300CMP membranes exhibited the highest Li^{+} permeation rate at approximately 4×10^{-3} m/h, while the 5c50CMP membranes displayed the lowest Li^{+} permeation rate of 5×10^{-5} m/h (Supplementary Figure 35). This considerable range in tuning ion permeation rates underscores the efficacy of electropolymerization as a potent technique for graphene repair and the engineering of graphene defects.

To further confirm the role of nanoporous graphene on the ion sieving, a 6-second plasma-treated graphene (referred to as "6sGr") was used to prepare the control sample. The pore size of the graphene treated by the 6-second plasma is around 2.4 nm on average as reported in our previous work[15]. This graphene was also masked by a CMP layer prepared under various electropolymerization conditions (5 CV cycles with a scan rate of 50 mV/s, 200 mV/s, and 300 mV/s, respectively). As shown in Supplementary Figure 37, the 6sGr membrane (supported by CNT film and without CMP masked layer) exhibited a high ion permeation rate with a Li^{+}/Mg^{2+} selectivity of ~1. It indicates that 6 seconds of plasma-treated graphene loses the Li^{+}/Mg^{2+} selectivity due to the excessive etching of the graphene and thus the large pores/defects in graphene. Compared with the CO_2 pore-expansion technique mentioned in the main text, the plasma method here cannot finely control the generation of similar-sized pores in graphene. After using the CMP polymer layer to mask the 6sGr, the membranes (CMP-masked 6sGr) prepared under various electropolymerization conditions did not show satisfactory ion selectivity. The highest Li^{+}/Mg^{2+} selectivity is lower than 3 as shown in Supplementary Figure 37. This is because the large pores in graphene (e.g., 3 nm) even masked by CMP still have

sizes equivalent to that in CMP. Hence, such pores will not be selective. This result indicates the significance of the nanosized pores in graphene to the ion sieving performance.

In summary, the results shown in Supplementary Figures 35 and 37 indicate that the synergistic effects between the CMP mask layer and the nano-sized pores in Gr play an important role in sieving ions.

Supplementary Note 14

Effects of the CMP thickness of CMP-masked Gr on the ion sieving performance

The effect of the thickness of the CMP mask layer on ion sieving was investigated (Supplementary Figure 38). The CMP layers with three different thicknesses prepared using 3, 5, and 10 CV cycles, at the scan rate of 200 mV/s, were used to mask porous graphene (5minCO_2-Gr). The estimated thickness of these CMP mask layers for the three CV cycles is 6, 10, and 20 nm, respectively. The obtained membranes were referred to as "3cCMP-masked Gr", "5cCMP-masked Gr", and "10cCMP-masked Gr", respectively. 3cCMP-masked Gr exhibited a similar Li^+/Mg^{2+} selectivity to that of the porous Gr without CMP, around 17, suggesting negligible mask effects of the CMP layer to the large pores in the graphene. One potential explanation could be that the CMP layer obtained under 3 CV cycles is either excessively thin or discontinuous, rendering it inadequate in masking the large pores and defects in the graphene. Consequently, it cannot be successful in significantly enhancing ion selectivity as intended. On the other hand, 10cCMP-masked Gr showed a significant decrease in both ion selectivity and ion permeation rate. This suggests that an excessively dense CMP layer with significant thickness on porous graphene could impede the diffusion rate of Li^+, primarily due to the substantial resistance encountered in mass transfer. As a result, both Li^+ ions and Mg^{2+} ions are unable to rapidly cross the membrane, resulting in the inability to achieve high selectivity. Ultimately, the CMP film prepared with 5 CV cycles under a scan rate of 200 mV/s was selected as the optimal mask layer.

Supplementary Note 15

Effects of concentration on ion sieving performance

The ion separation performance of the CMP-masked Gr membranes driven by different salt concentrations was compared (Supplementary Figure 40). The results show that there is not a significant difference in normalized ion permeation rate (with an average value of 5.03×10^{-4} for Li^{+} and 2.60×10^{-7} Mg^{2+}) and ion selectivity tested under different concentrations. This may be because we have a sufficiently fast stirring by a magnetic stirrer and the volume of the diffusion cell is large enough that attenuate the effects of the concentration polarization.

Supplementary Note 16

Ion sieving performance under electric field-driven process

The electric field-driven ion diffusion tests (Supplementary Figures 44 and 45) were employed to assess the ion separation performance of the CMP-masked Gr membranes. The results reveal that Li^+ ions displayed a significantly faster permeation rate due to their smaller size compared to the divalent ions Ca^{2+} and Mg^{2+}. The calculated conductance of Li^+ is $5.2 \pm 0.7 \times 10^{-2}$ mS, approximately 30 times higher than that of Mg^{2+}. It suggests the great potential in electric field-driven applications. This transport property aligns with observations from the concentration-driven permeation tests. It is noteworthy that the ion selectivity tested under the electric field-driven process may not be very close to that tested under the concentration-driven process. This discrepancy is likely explained by the forced migration of large ions due to partial dehydration when an electric field is applied, as reported in many studies[16-18].

Supplementary Supplementary Figures

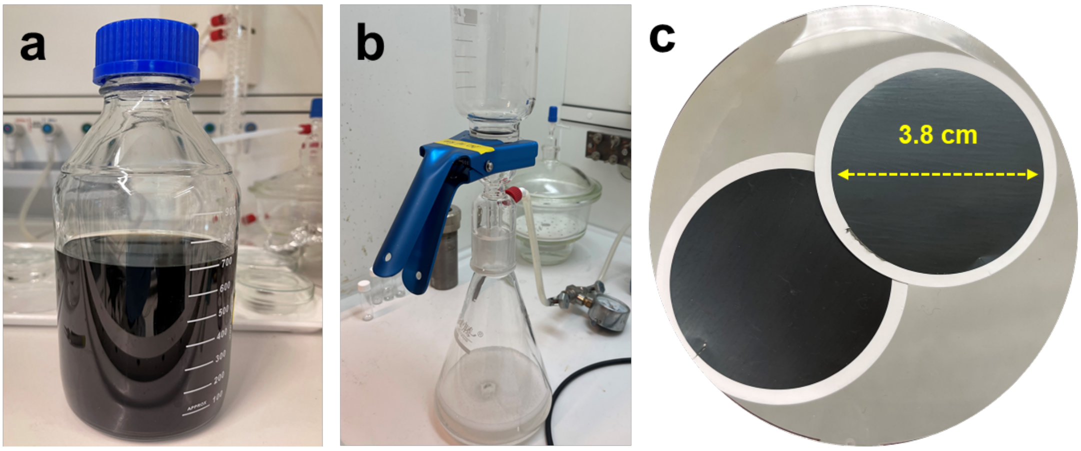

Supplementary Figure 1 Optical photographs of a) the CNT solution, b) the vacuum filtration setup, and c) CNT/PES membranes.

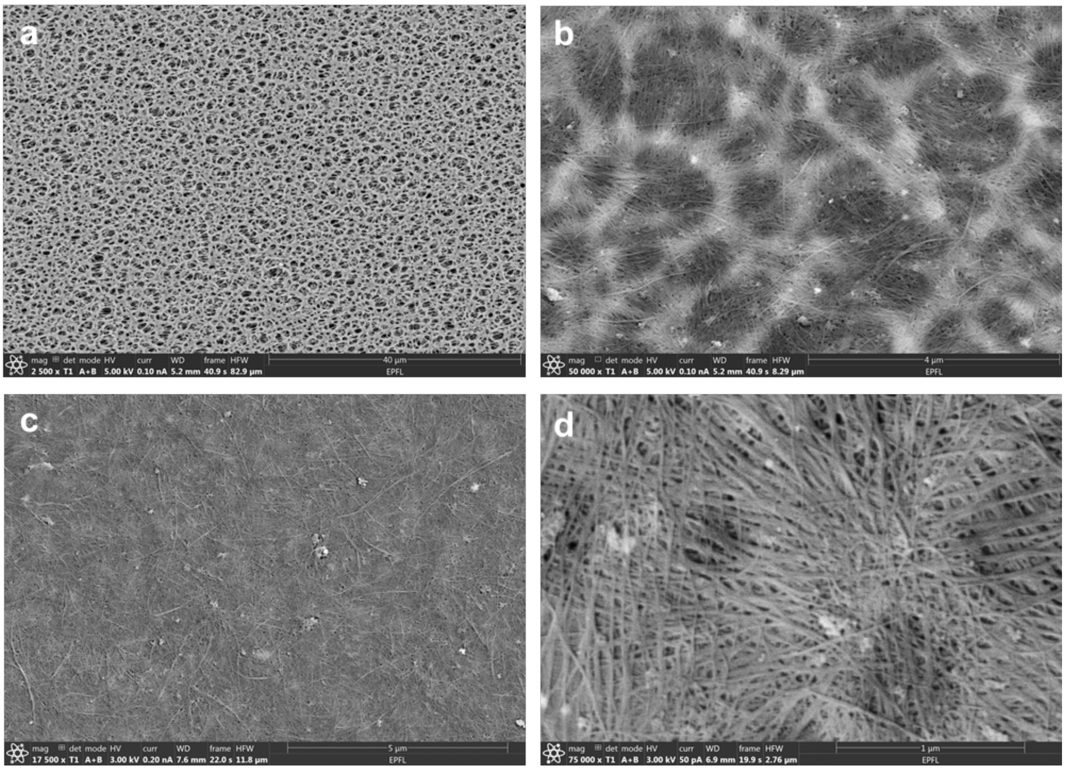

Supplementary Figure 2 Morphology of the CNT-PES support. a) Surface SEM image of the pristine PES substrate, b) surface SEM image of the CNT-PES support (filtrating with 10 mL CNT solution on the PES substrate), c) and d) surface SEM images of the CNT-PES support (filtrating with 10 mL CNT solution on the PES substrate).

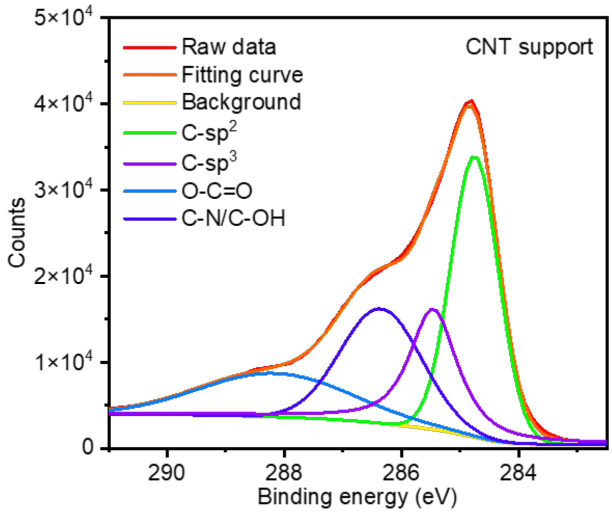

Supplementary Figure 3 C1s XPS spectra from the free-standing CNT film.

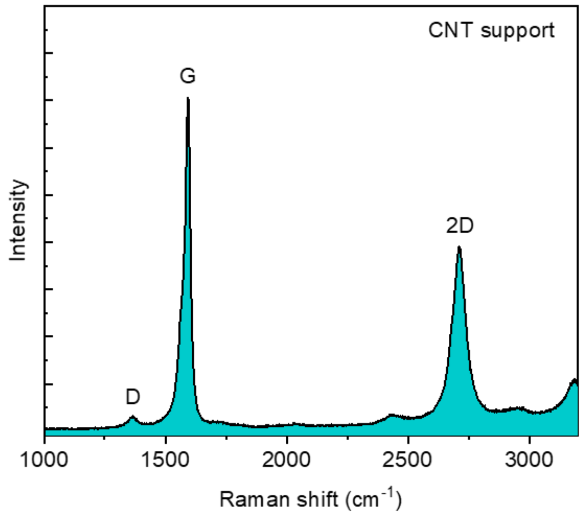

Supplementary Figure 4 Raman spectra of the free-standing CNT film.

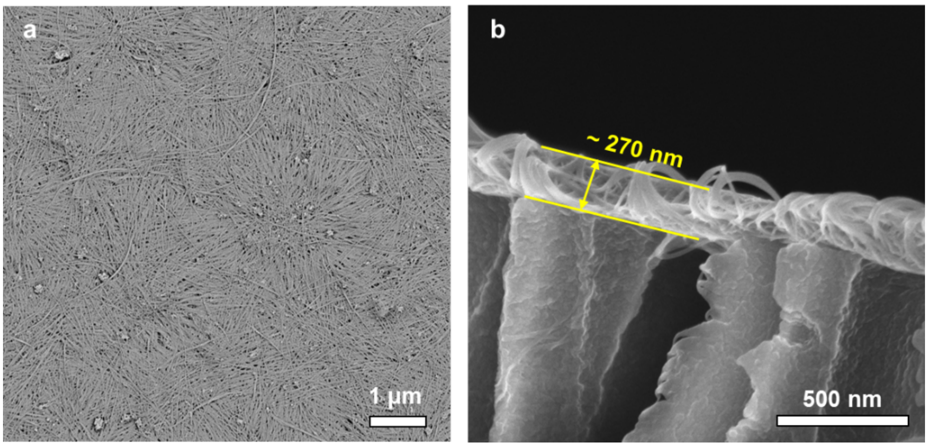

Supplementary Figure 5 Morphology of the CNT free-standing film. a) Surface SEM image of the CNT free-standing film, b) cross-sectional SEM image of the CNT film.

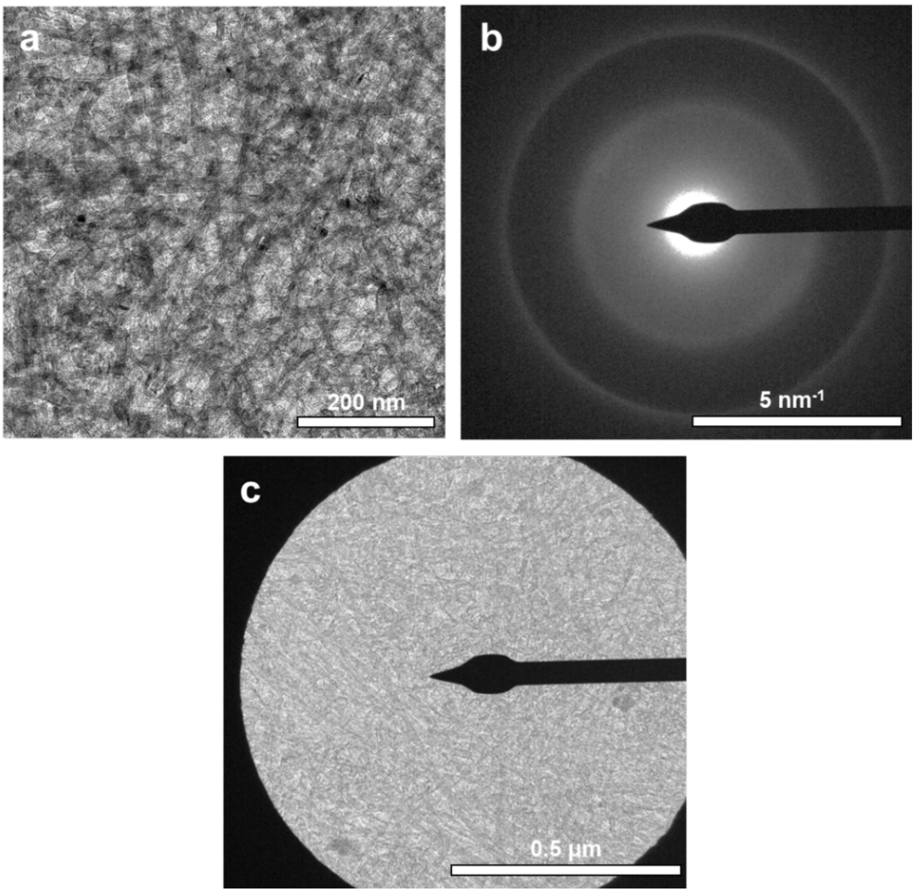

Supplementary Figure 6 Morphology of the free-standing CNT film. a) TEM image of the CNT free-standing film, b) a SAED pattern from the region shown in c) of the free-standing CNT film.

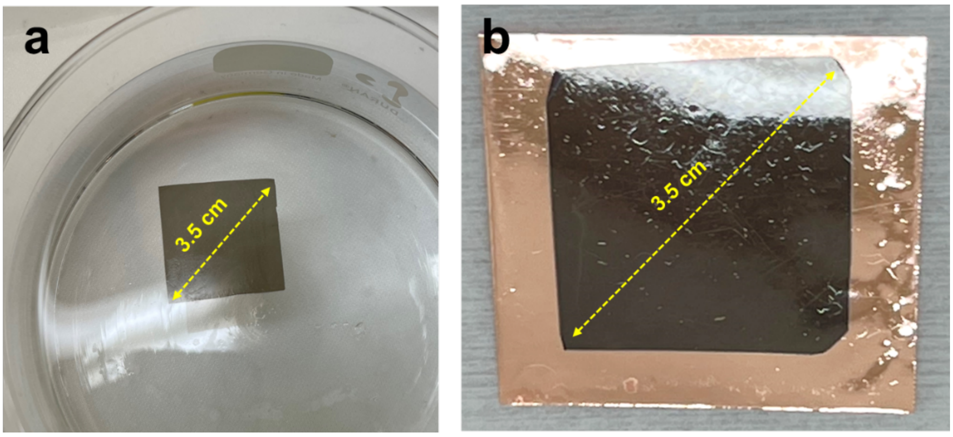

Supplementary Figure 7 Optical photographs of a) the free-standing CNT film on water and b) CNT/graphene/Cu.

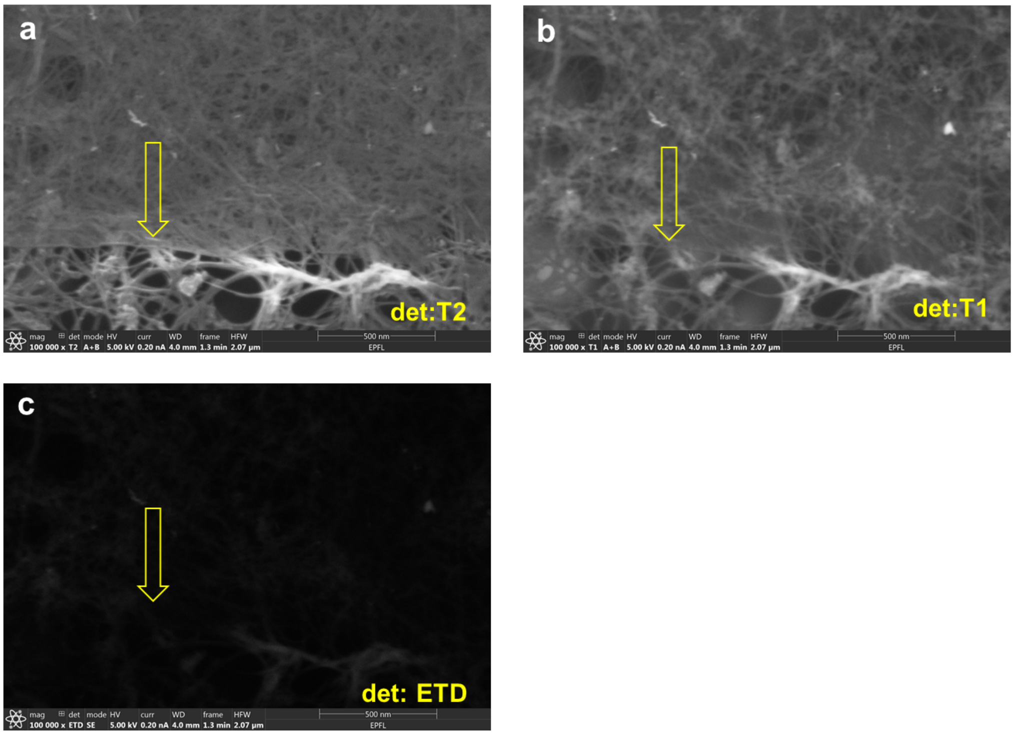

Supplementary Figure 8 Morphology of the CNT-supported monolayer graphene under a) backscattered electron detector, b) secondary electron detector, and c) Everhart–Thornley detector. Graphene edge at the end of the film can be visualized here.

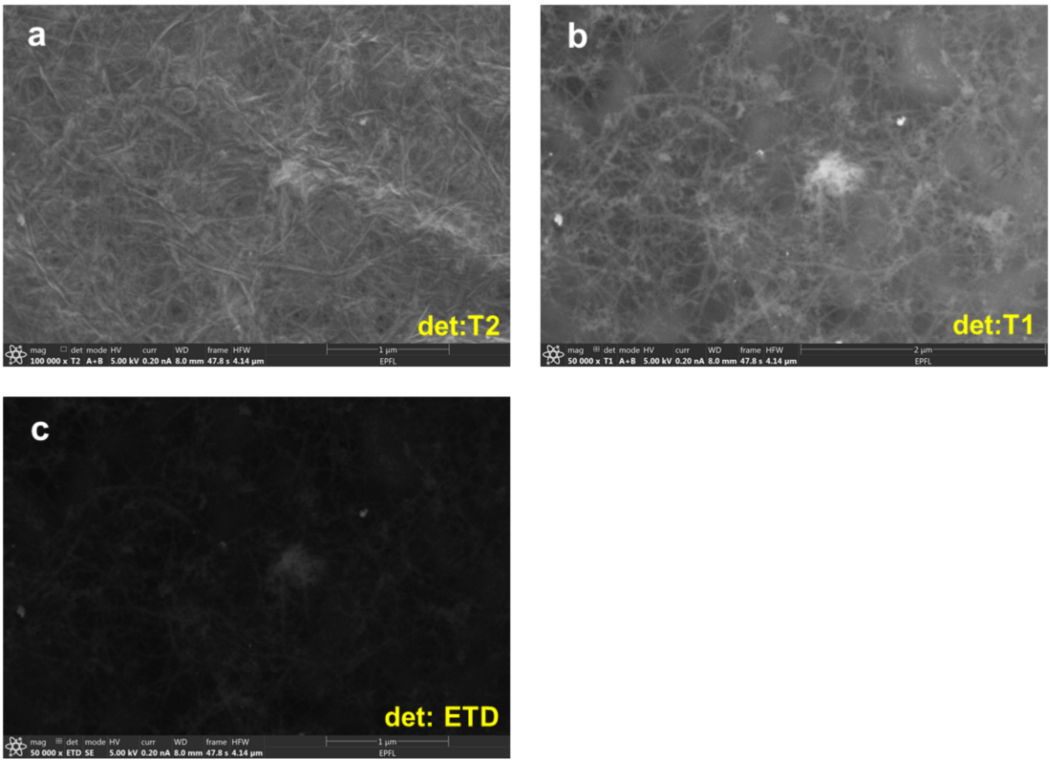

Supplementary Figure 9 Morphology of the CNT-supported monolayer graphene under a) backscattered electron detector, b) secondary electron detector, and c) Everhart–Thornley detector.

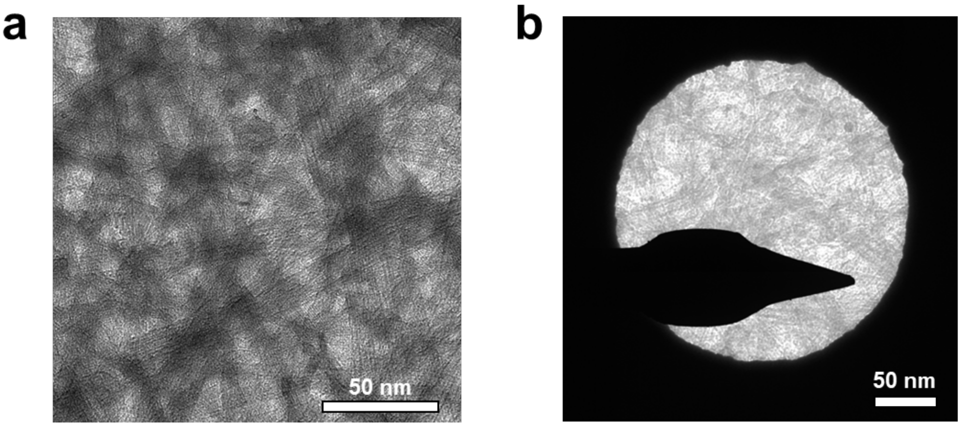

Supplementary Figure 10 TEM images of the CNT-supported graphene.

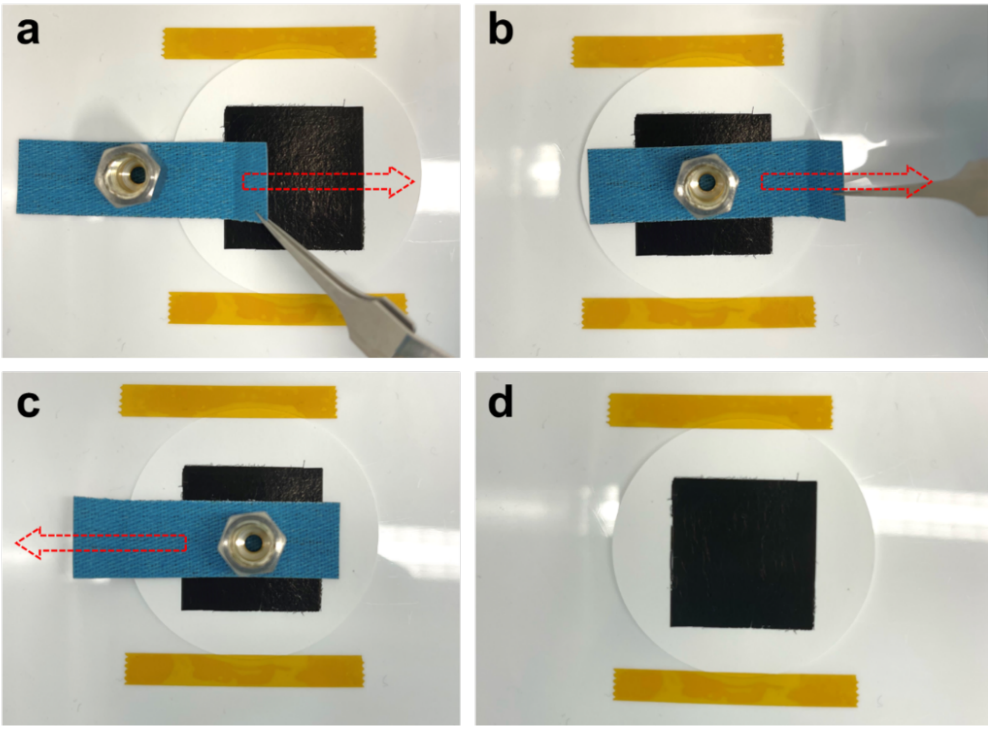

Supplementary Figure 11 Mechanical stability of a CNT-Graphene membrane. P2000 sandpaper scrubbing on the membrane with a weight of 6.4g was loaded on the sandpaper.

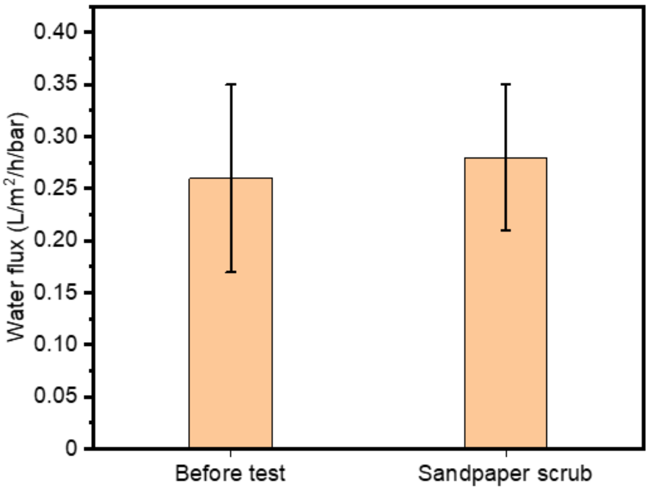

Supplementary Figure 12 Water flux of the membrane before and after the abrasion tests.

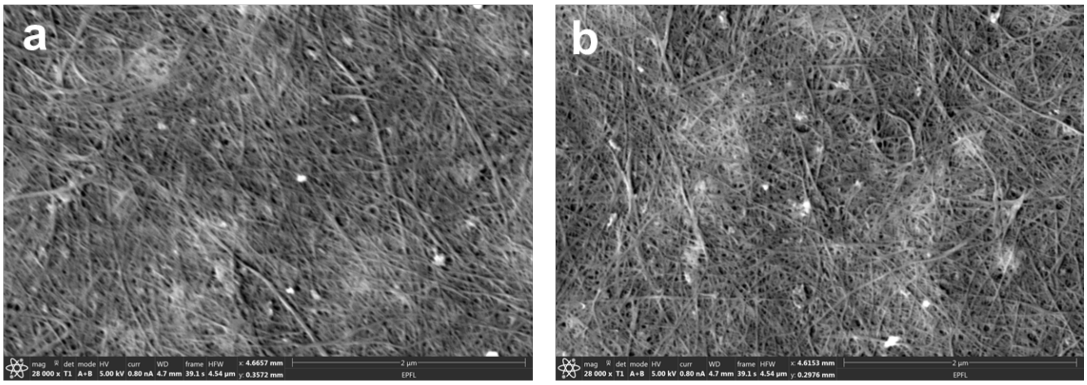

Supplementary Figure 13 SEM images of the membrane a) before and b) after the abrasion tests.

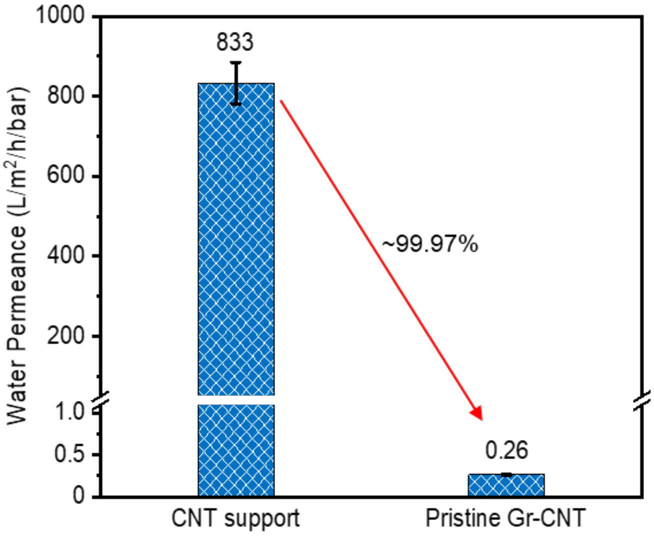

Supplementary Figure 14 Water permeance of the CNT support and the CNT-supported pristine graphene.

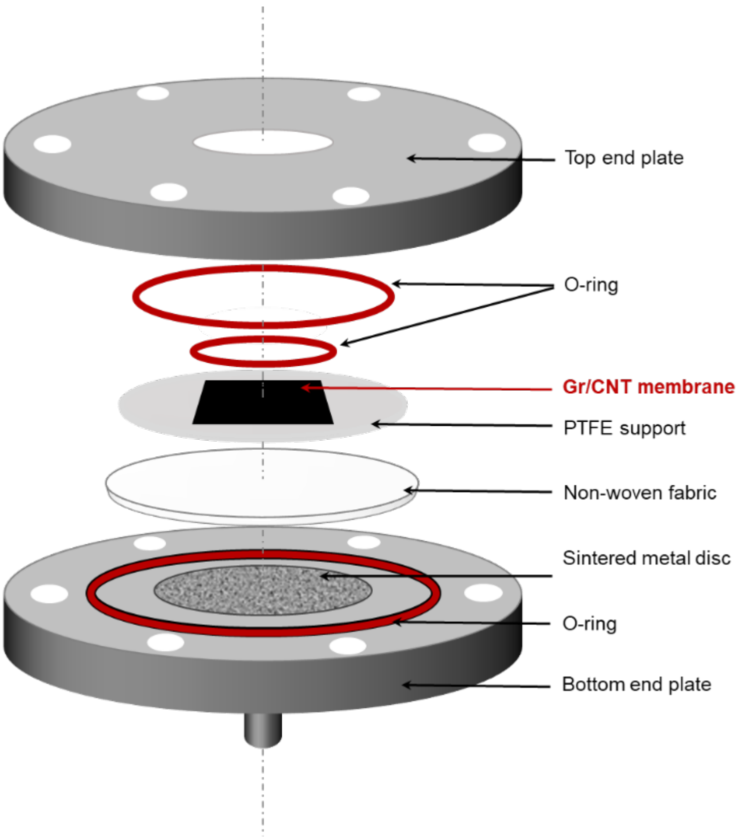

Supplementary Figure 15 Schematic diagram of the membrane module used in the pressure-driven nanofiltration performance tests.

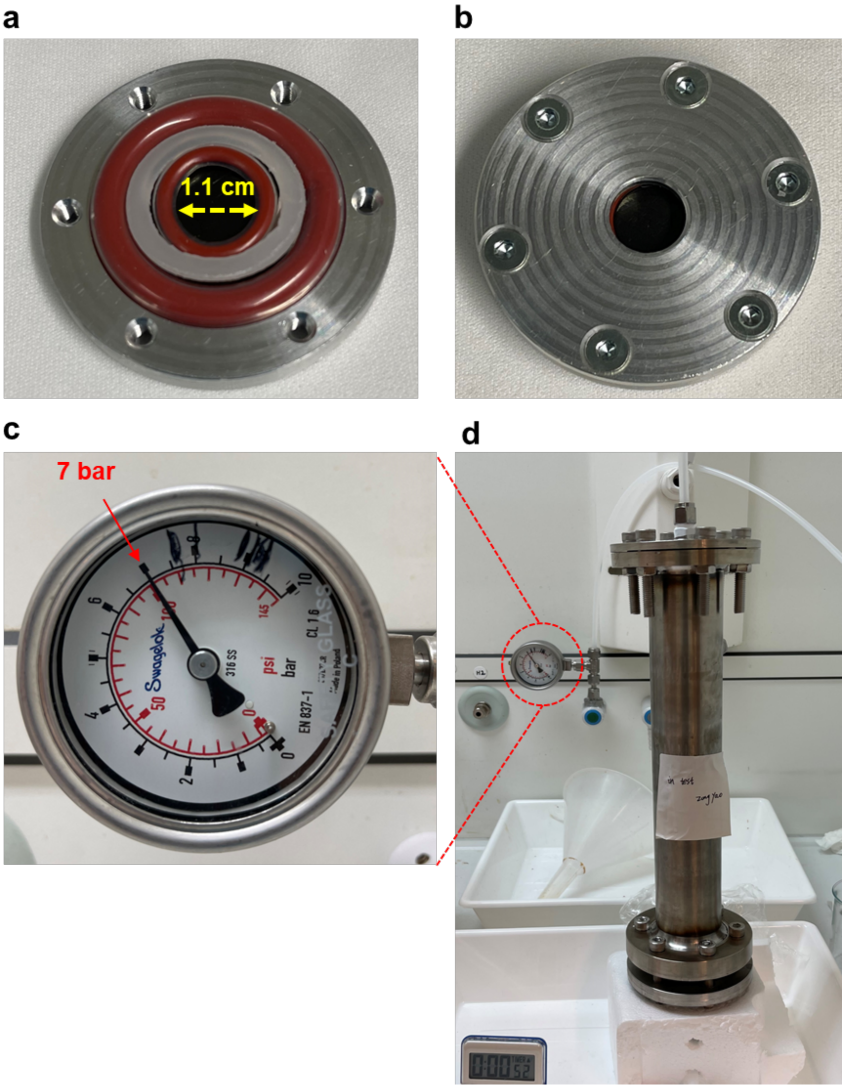

Supplementary Figure 16 Optical photographs of a) the membrane module and b) the sealed membrane, c) and d) optical photographs of the setup used in the pressure-driven nanofiltration performance tests.

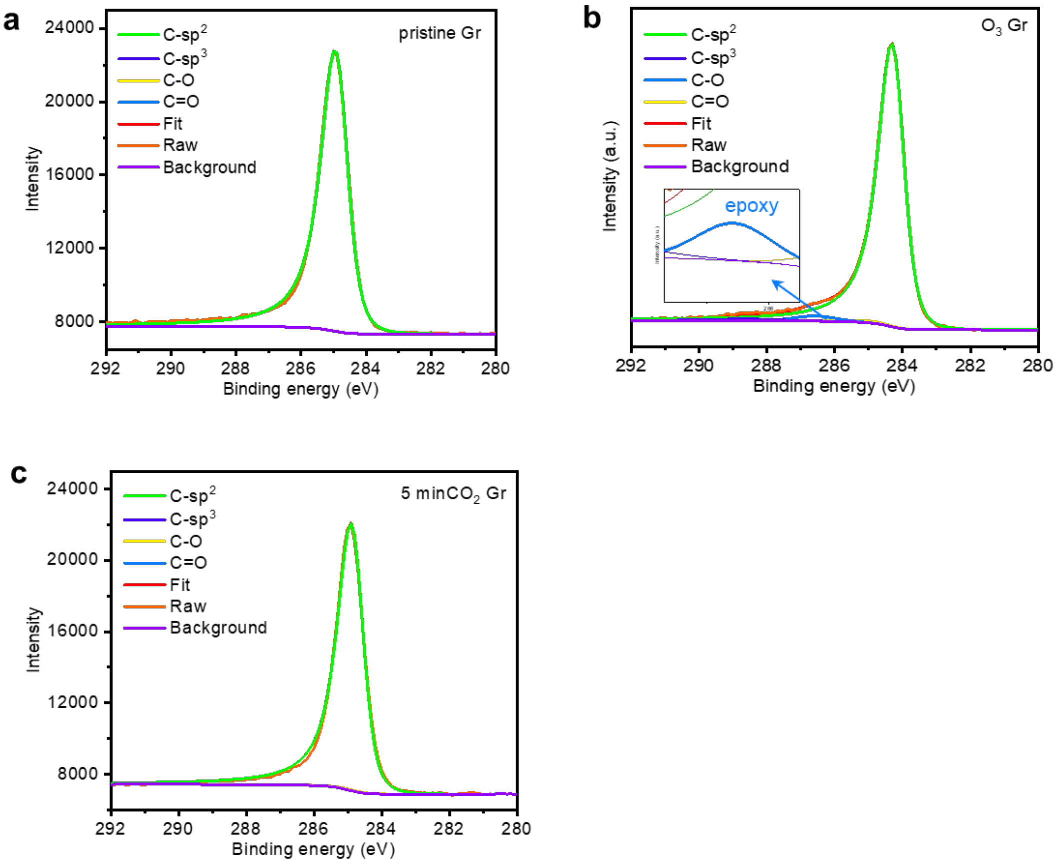

Supplementary Figure 17 C1s XPS spectra of a) pristine graphene, b) O_3-treated graphene, and c) CO_2-treated graphene.

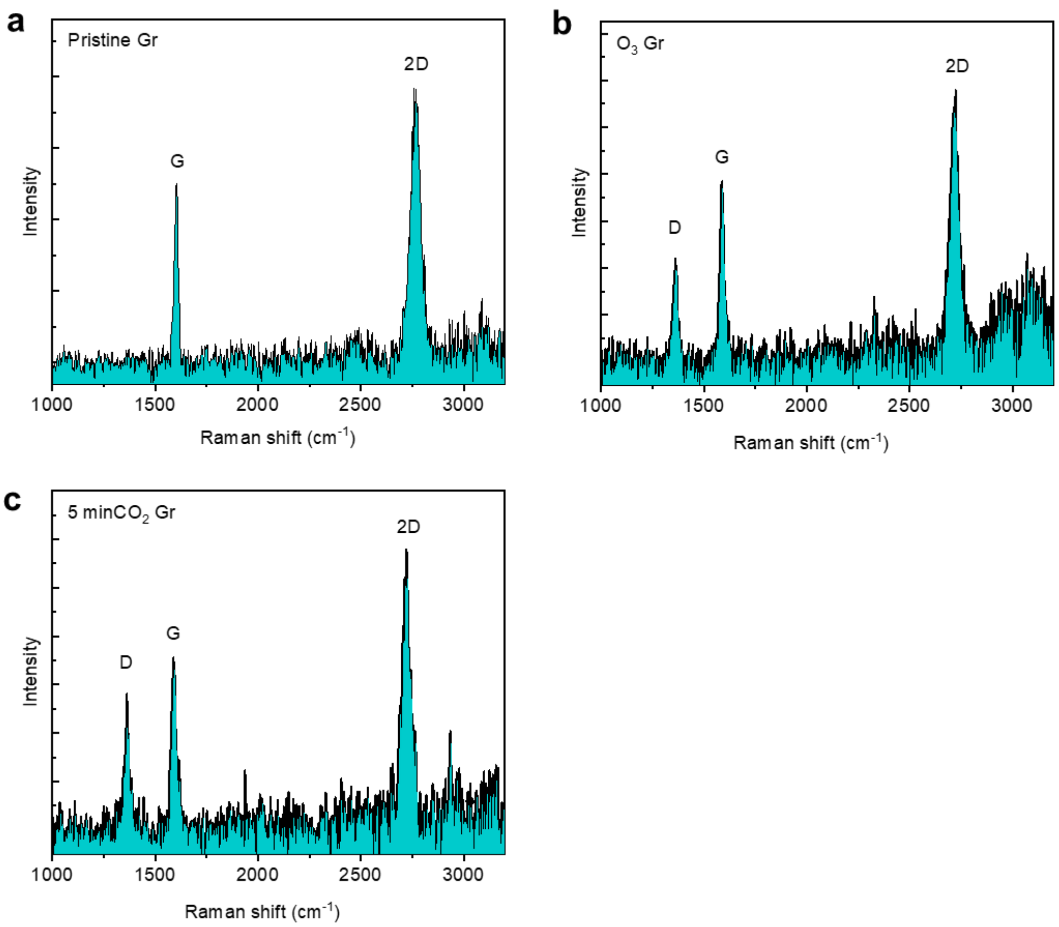

Supplementary Figure 18 Raman spectra of a) pristine graphene, b) O_3-treated graphene, and c) CO_2-treated graphene.

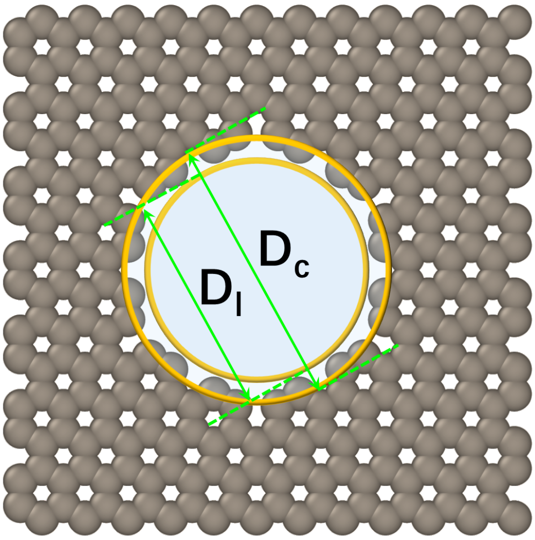

Supplementary Figure 19 Schematic of the measurement of the pore size in the graphene.

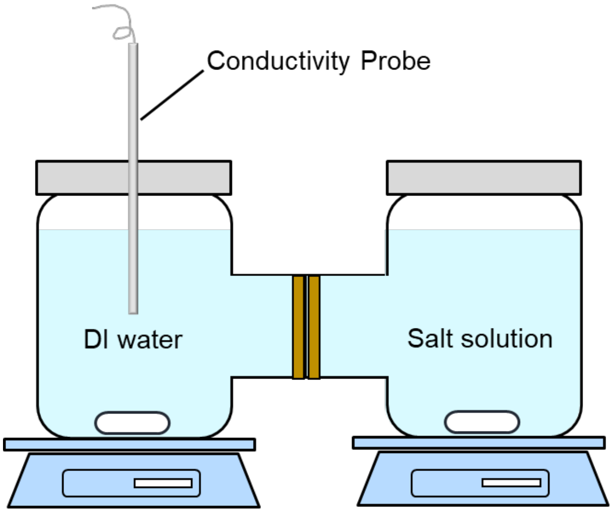

Supplementary Figure 20 Schematic diagram of the setup used in the concentration-driven performance tests.

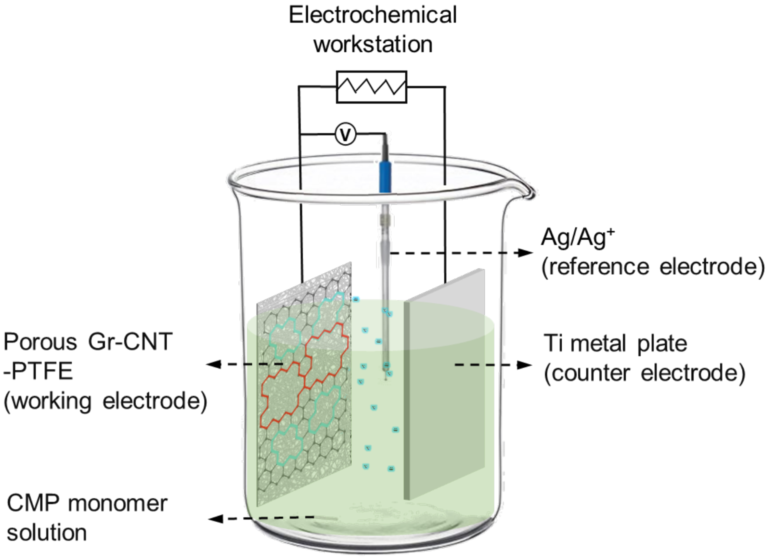

Supplementary Figure 21 Schematic of the electropolymerization device.

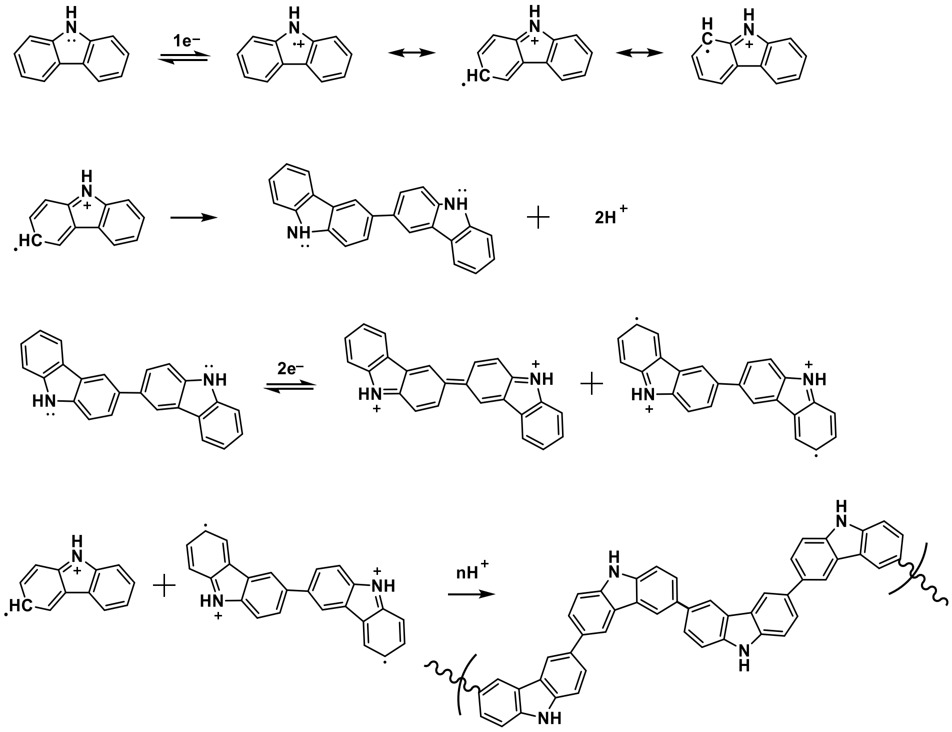

Supplementary Figure 22 Mechanism of electropolymerization of CMP mask layer.

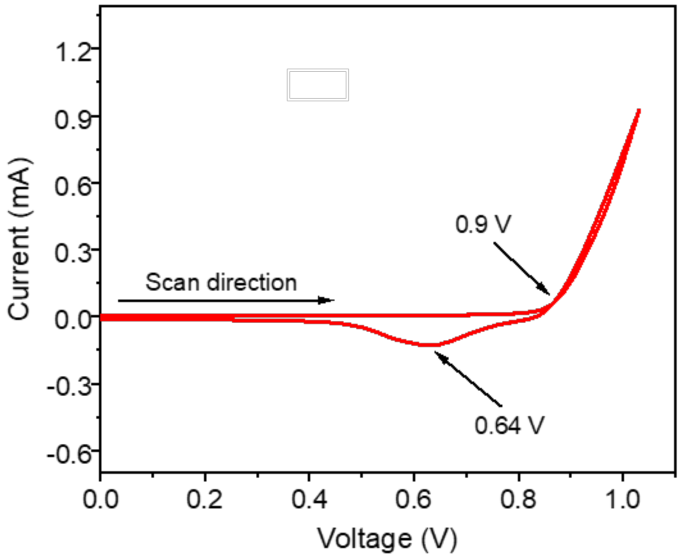

Supplementary Figure 23 Current curves recorded in the first CV cycle.

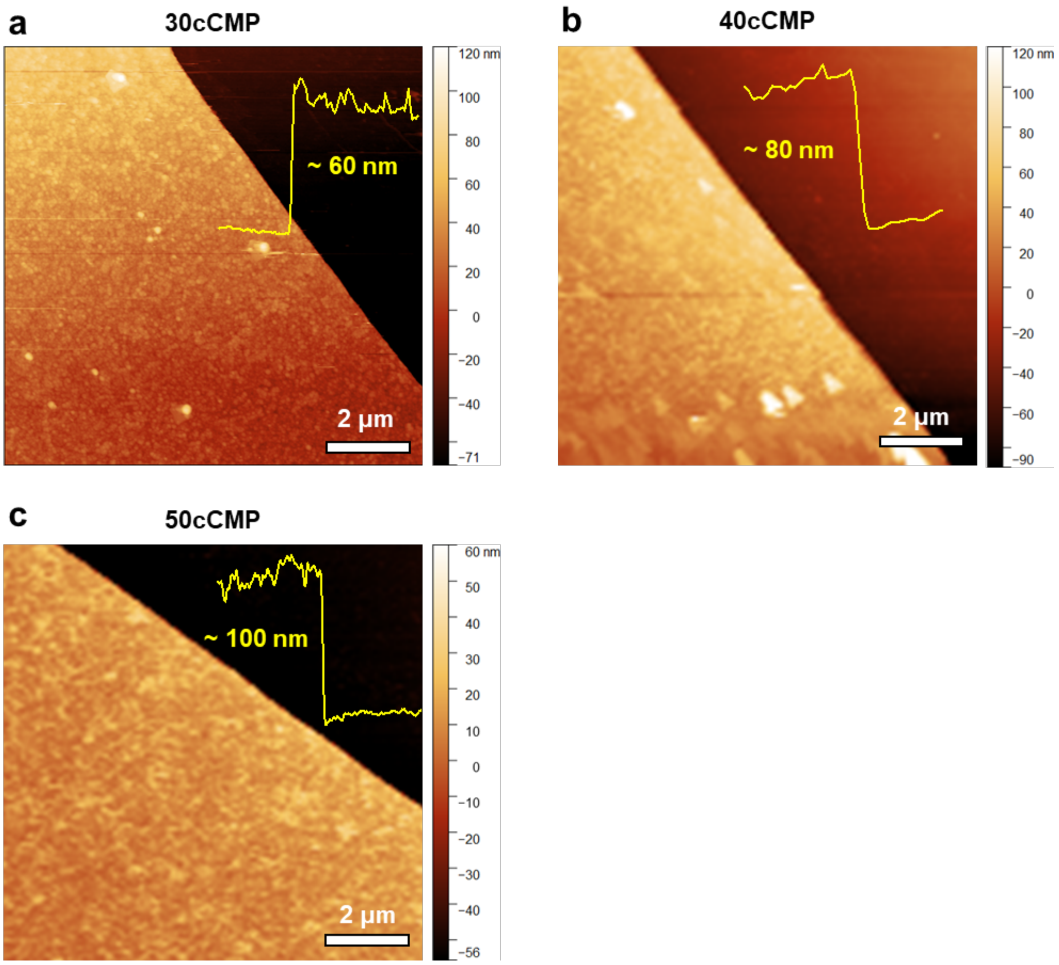

Supplementary Figure 24 AFM images of the CMP films prepared with a) 30 CV cycles, b) 40 CV cycles, and c) 50 CV cycles, at the scanning rate of 200 mV/s.

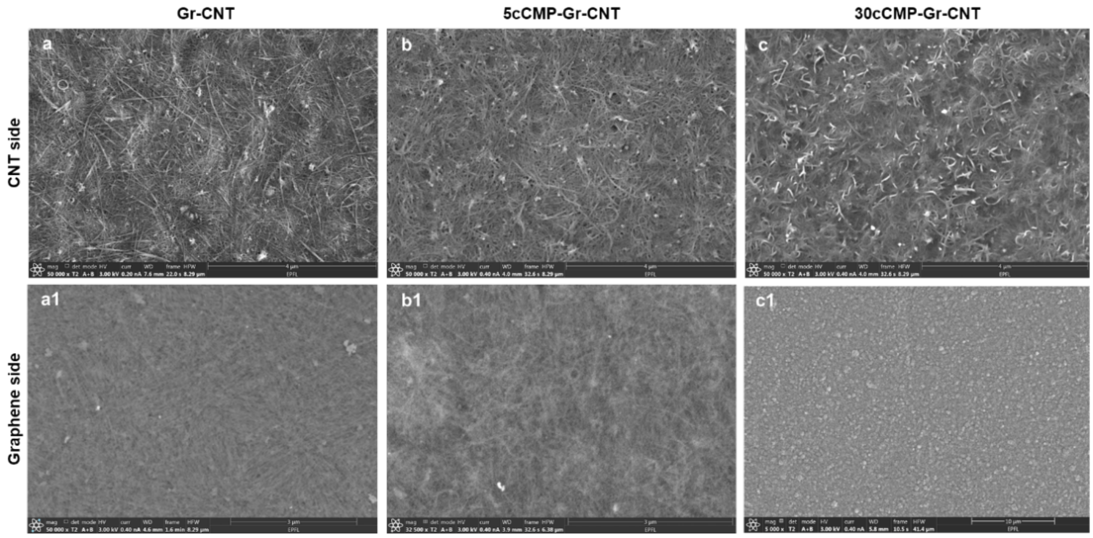

Supplementary Figure 25 Surface SEM images of the top and back sides of the membranes. a) CNT side and a1) graphene side of the CNT-supported graphene, b) CNT side and b1) graphene side of the CMP-masked (5 CV cycle) porous graphene, and c) CNT side and c1) graphene side of the CMP-masked (30 CV cycle) porous graphene.

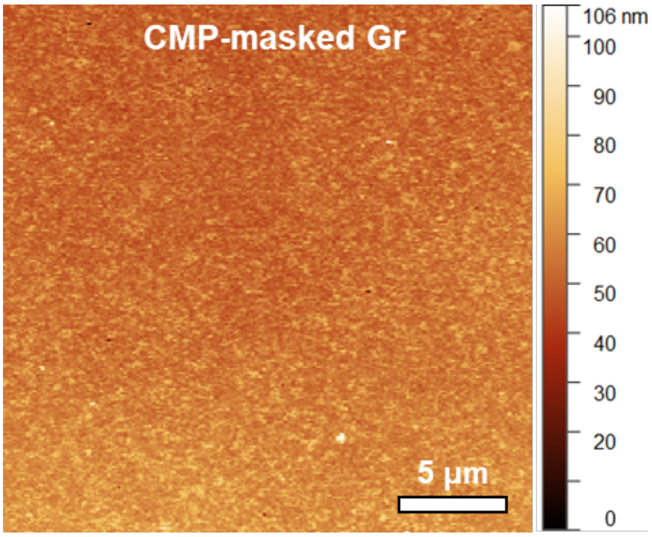

Supplementary Figure 26 AFM image of the 5cCMP-Gr membrane

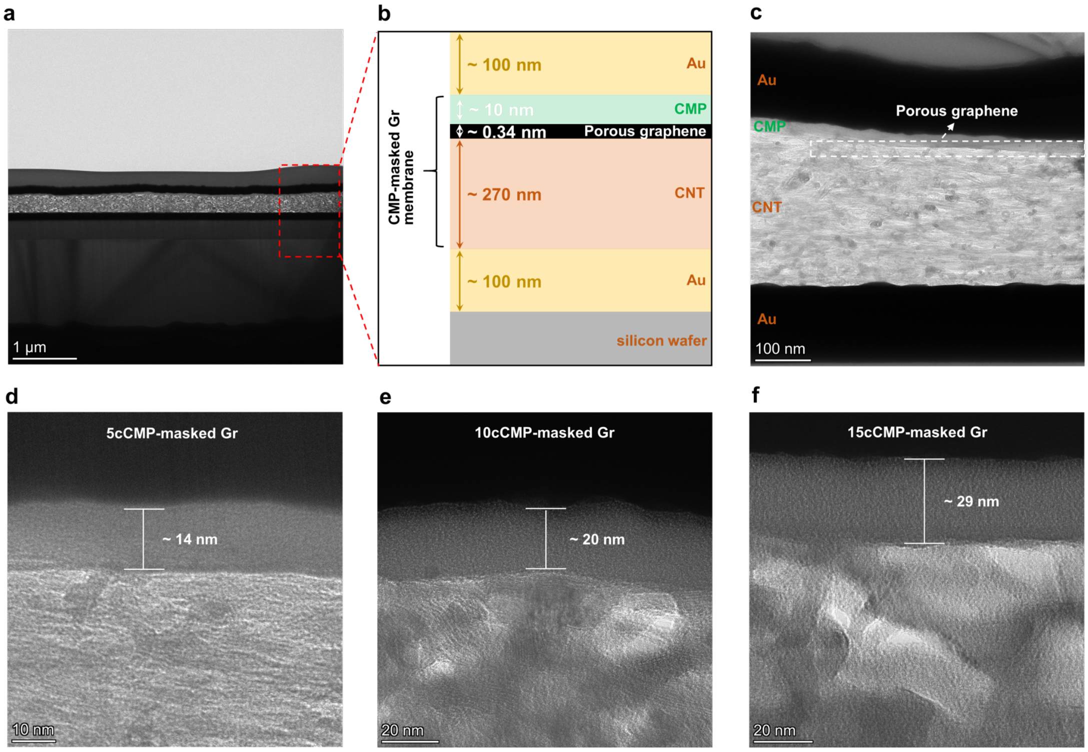

Supplementary Figure 27 a) and c) Cross-section TEM images of the CMP-masked Gr membranes, b) schematic structures of the membrane, d), e), and f) cross-section TEM images of the CMP-masked Gr membrane prepared under different CMP synthesis conditions (with 5, 10, and 15 CV cycles, at the scanning rate of 200 mV/s).

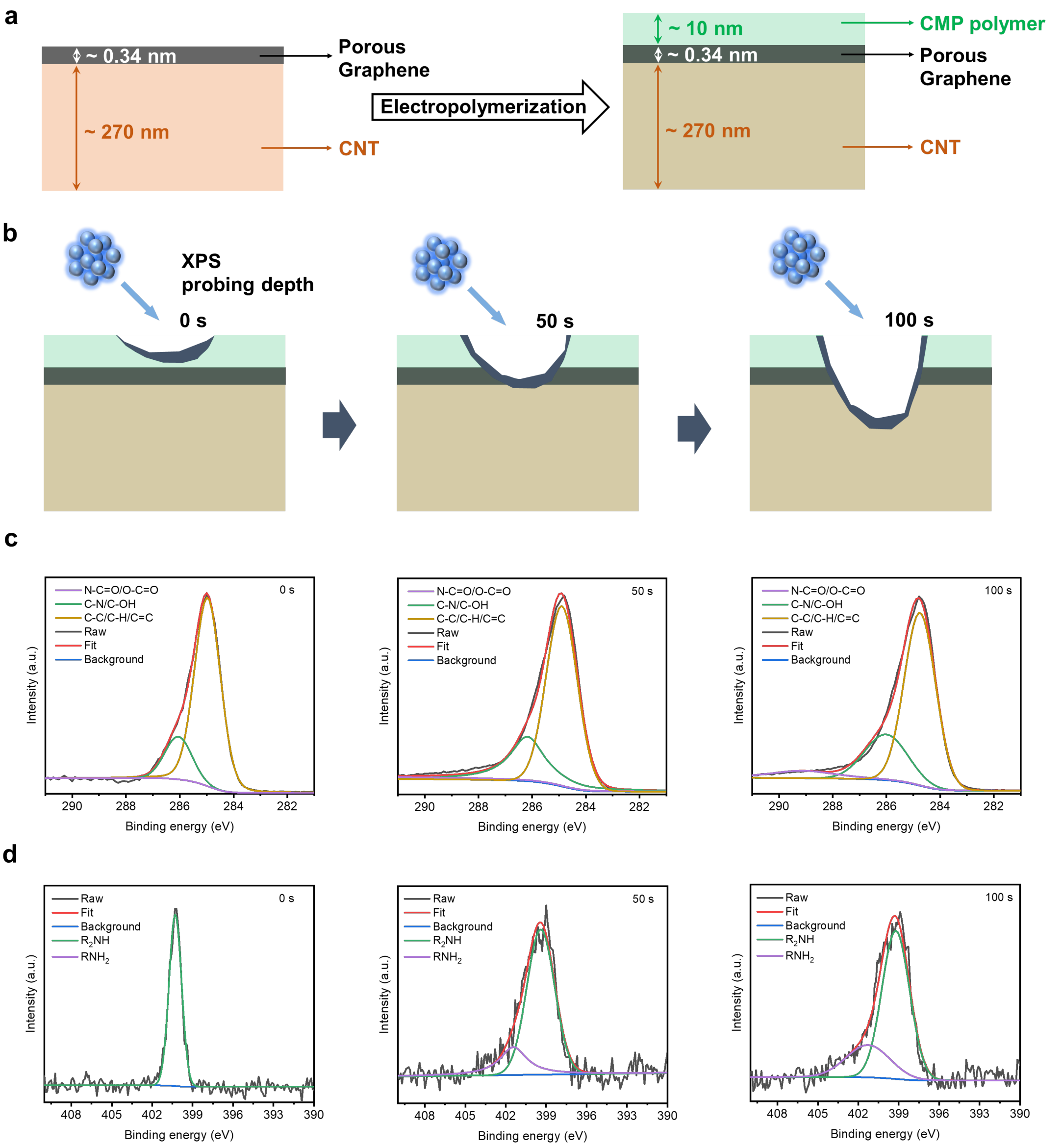

Supplementary Figure 28 XPS depth profile analysis. a) Schematic structures of the membrane, b) schematic structures for the XPS depth-profiling of the membrane, c) evolution of C1s peaks as a function of sputter time, and d) evolution of N1s peaks as a function of sputtering time.

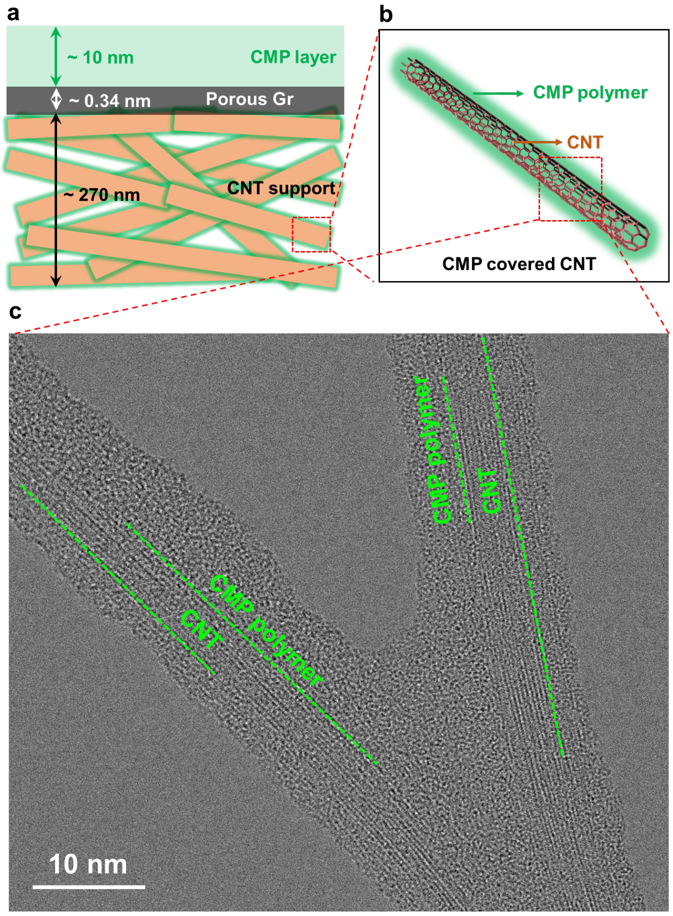

Supplementary Figure 29 a) Schematic structures of the membrane, b) schematic of CMP polymer covered CNT, and c) TEM image of CMP polymer covered CNT.

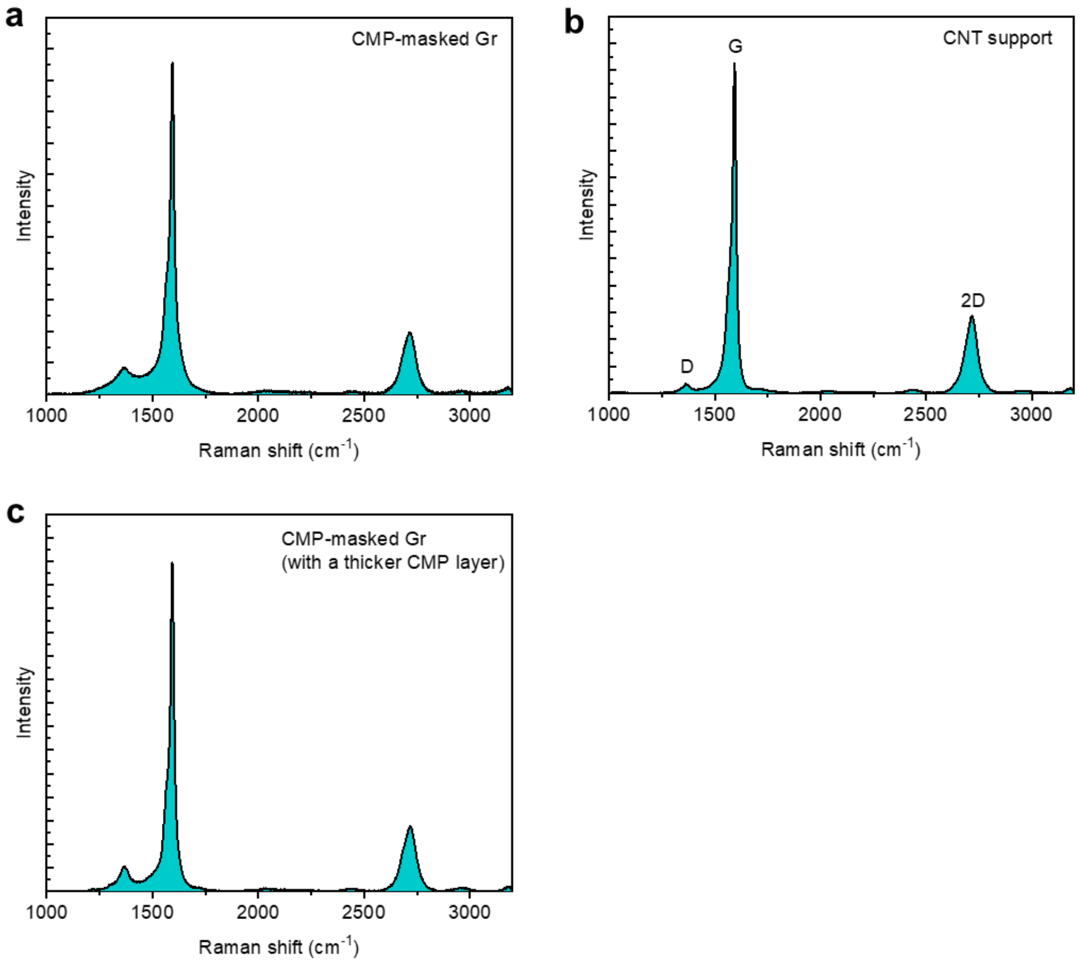

Supplementary Figure 30 Raman spectrums of CMP-masked Gr and CNT support.

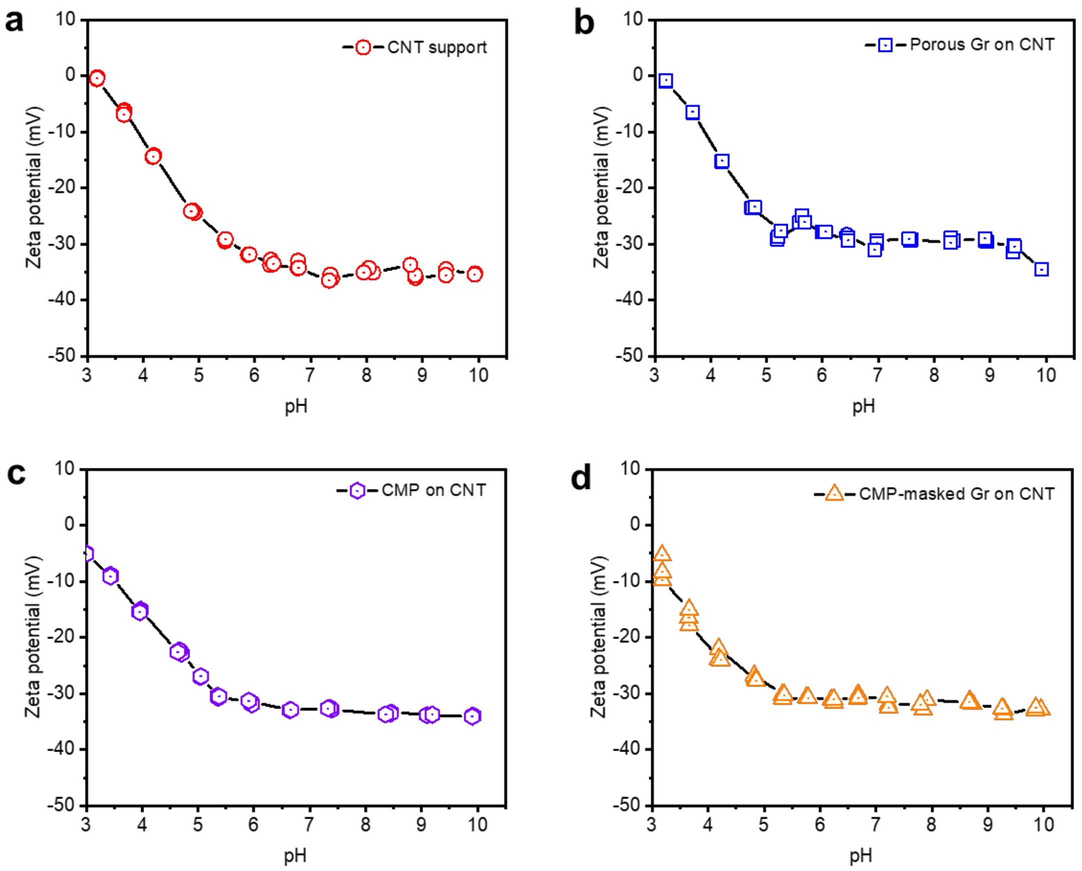

Supplementary Figure 31 Zeta potential of a) CNT support, b) porous Gr on CNT support, c) CMP on CNT support, and d) CMP-masked Gr on CNT support.

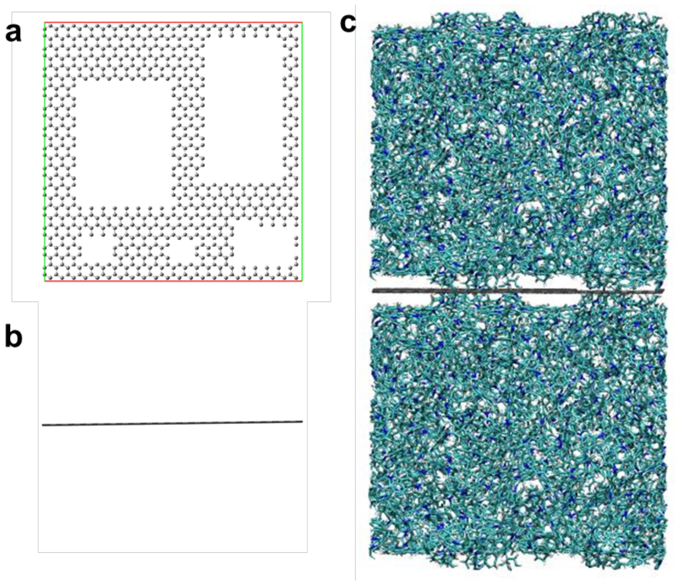

Supplementary Figure 32 Simulated structures: a) front view of the porous graphene with different size pores, b) side view of the porous graphene, and c) side view of the CMP-masked porous graphene membranes.

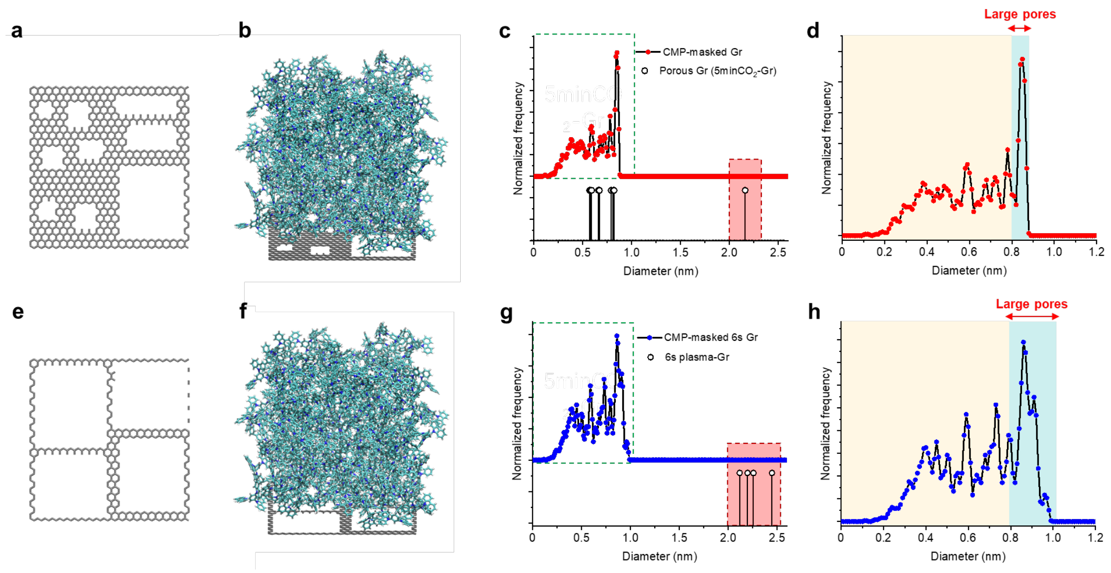

Supplementary Figure 33 Simulated structures and PSD: a) front view of the porous graphene (5minCO_2-Gr) with different size pores, b) side view of the graphene membrane with a CMP mask layer, c) simulated PSD of the CMP-masked Gr membrane and the sizes of the pores in the 5minCO_2-Gr before masking, d) a partially enlarged image of Supplementary Figure 33c), e) front view of the 6s plasma treated graphene (6s plasma-Gr) with four large pores, f) side view of the membrane with a CMP mask layer, g) simulated PSD of the CMP-masked 6s Gr membrane and the sizes of the pores in the 6s plasma-Gr before masking, and h) a partially enlarged image of Supplementary Figure 33g).

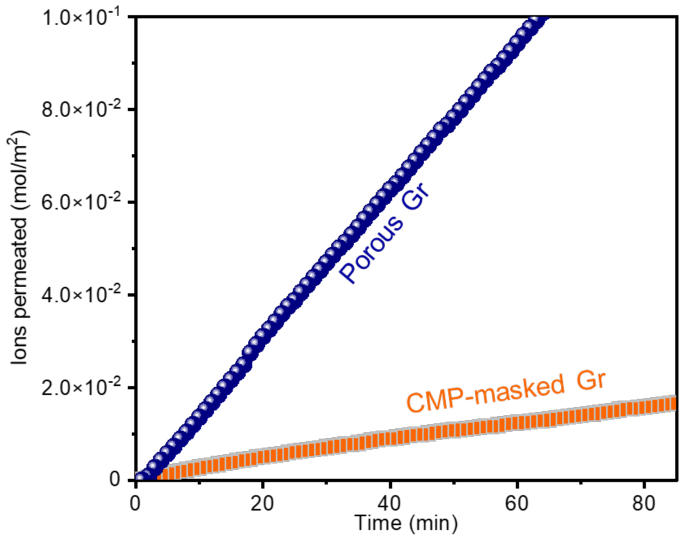

Supplementary Figure 34 Number of permeated Li^+ ions through porous graphene with and without CMP mask
layer.

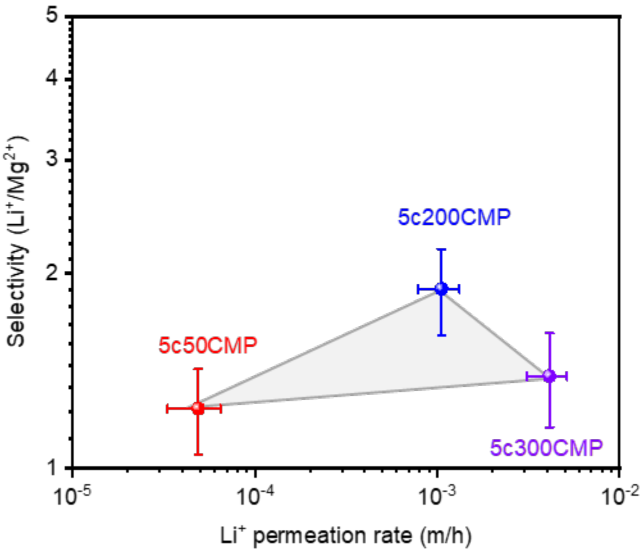

Supplementary Figure 35 Ion sieving performance of the CMP-CNT (without graphene) prepared under different scan rates (50, 200, and 300 mV/s) with 5 CV cycles.

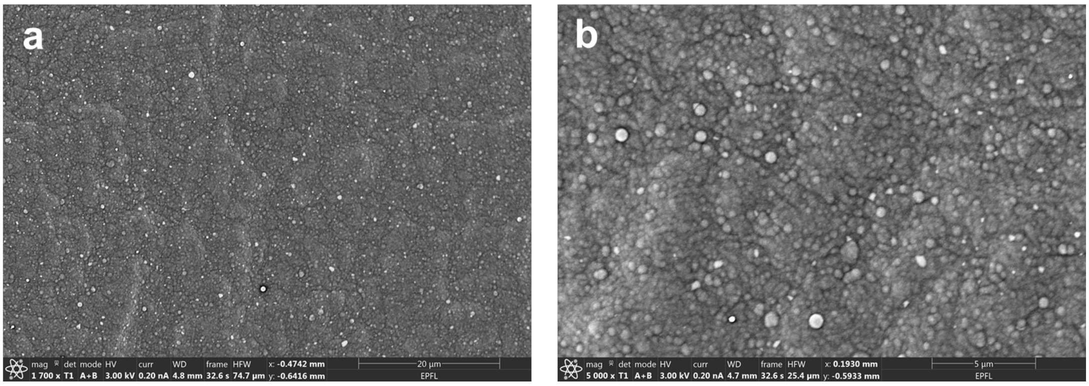

Supplementary Figure 36 SEM images of the membrane 5c50CMP (scan rates 50 mV/s, 5 CV cycles) on CNT support (without graphene) under different magnification.

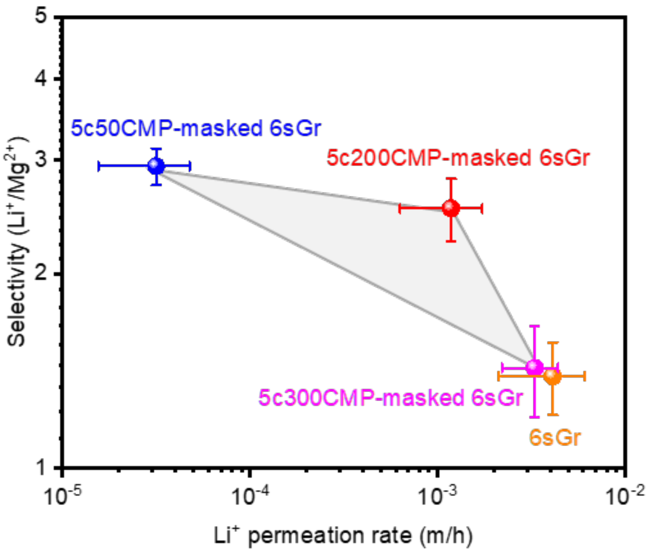

Supplementary Figure 37 Ion sieving performance of the CMP-masked graphene (treated by 6-second plasma) membranes. The CMP masking layers were prepared under different scan rates (50, 200, and 300 mV/s) with 5 CV cycles.

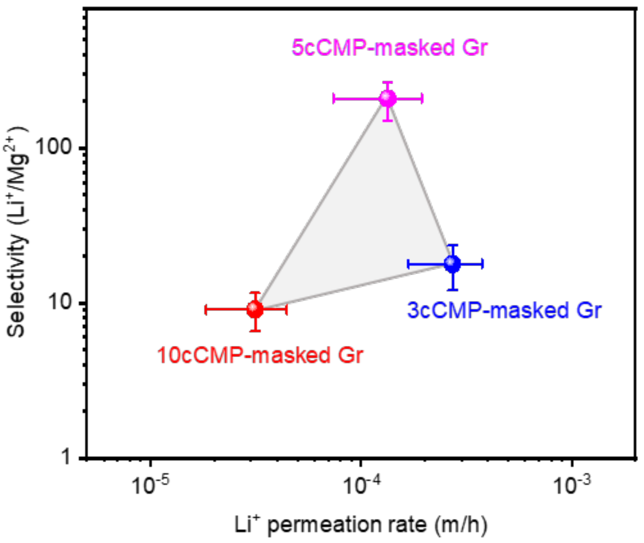

Supplementary Figure 38 Ion sieving performance of the CMP-masked graphene (treated by 5-minute CO_2) membranes with various CMP layers prepared with 3, 5, and 10 CV cycles under scan rate of 200 mV/s.

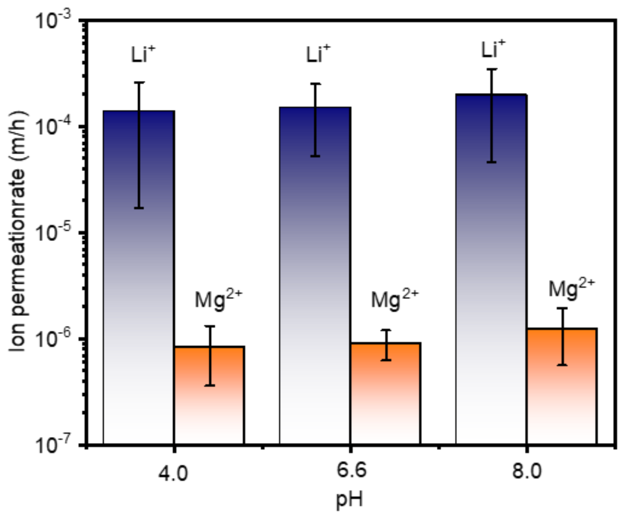

Supplementary Figure 39 Ion sieving performance as a function of pH.

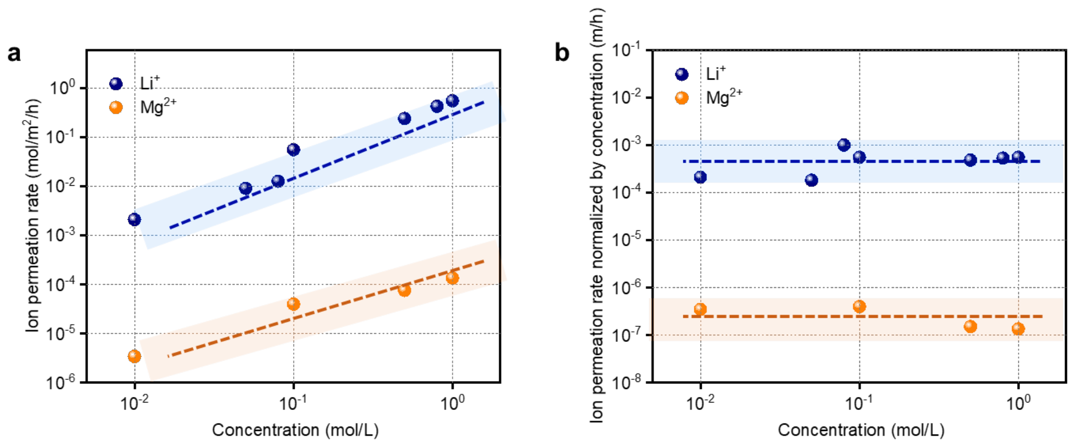

Supplementary Figure 40 a) Ion permeation rate as a function of salt solution concentration, b) ion permeation rate with unit normalized by the concentration difference.

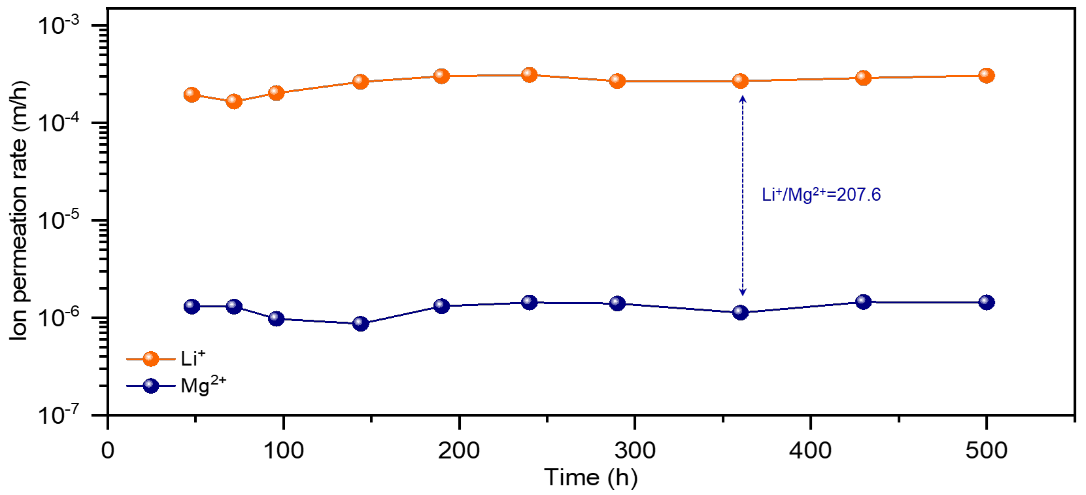

Supplementary Figure 41 Long-term binary ion sieving performance of the CMP-masked porous graphene.

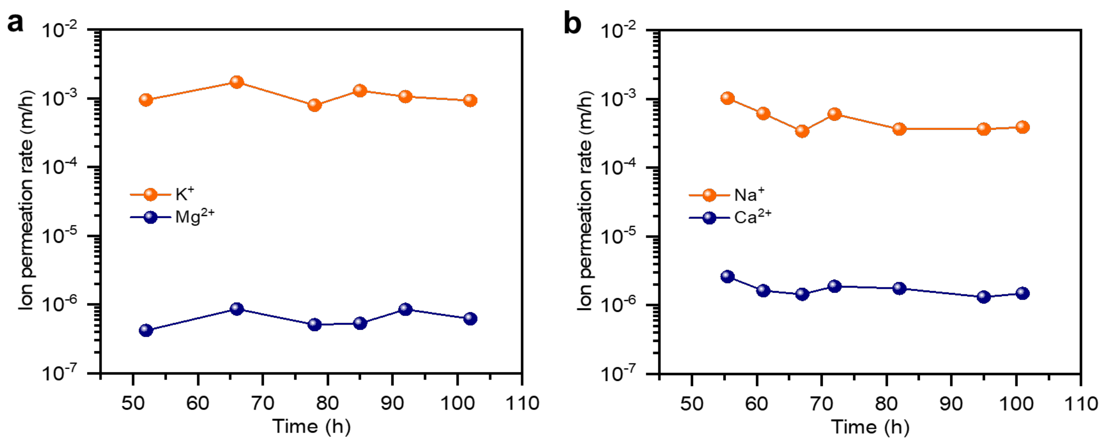

Supplementary Figure 42 Long-term a) K^+/Mg^{2+} and b) Na^+/Ca^{2+} binary ion sieving performance of the CMP-masked porous graphene membrane.

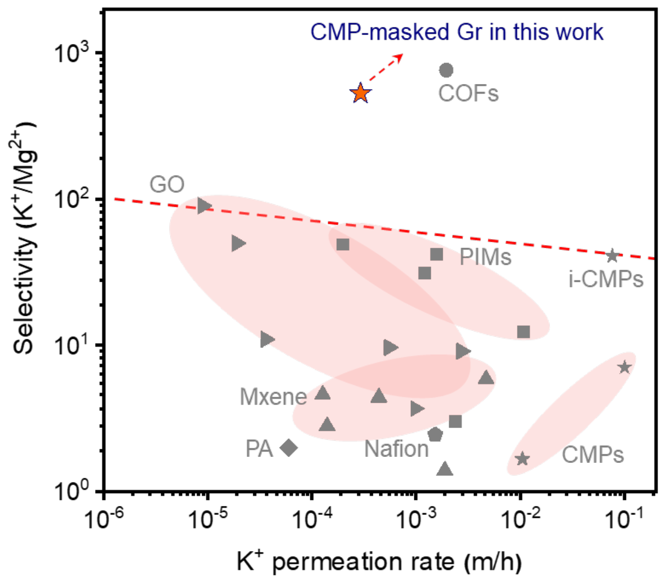

Supplementary Figure 43 Trade-off relationship between K^+/Mg^{2+}selectivity and K^+ permeation rate of the CMP-masked graphene membranes and the state-of-the-art ion-sieving membranes reported in the literature, tested under concentration-driven single-ion process. The red line was added manually to show the trade-off effect between ion permeation and selectivity.

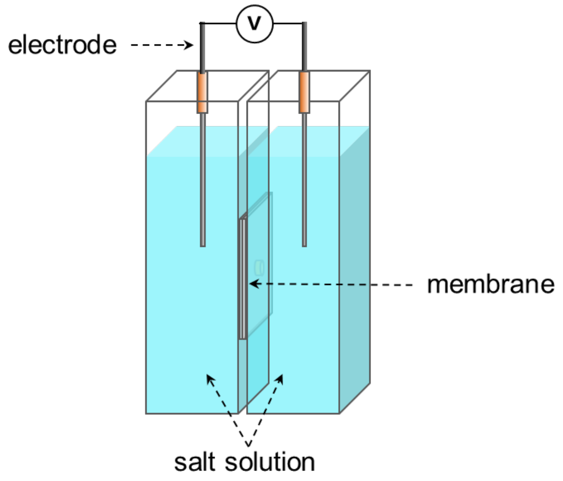

Supplementary Figure 44 Schematic of the setup for the ion sieving tests under electric field.

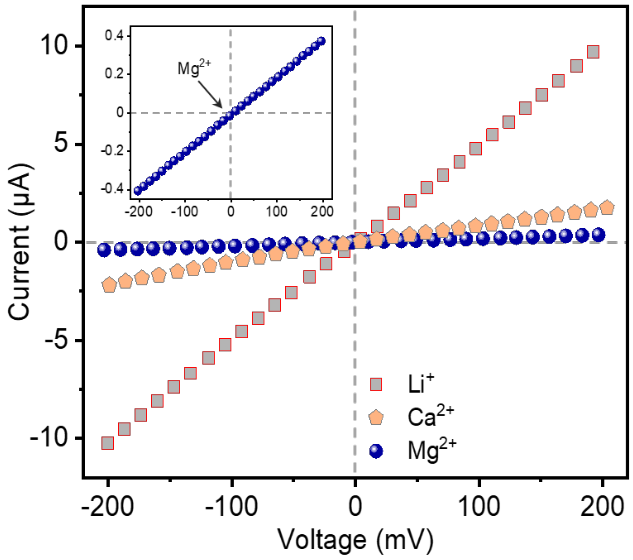

Supplementary Figure 45 Current-voltage (*I*-*V*) characteristics CMP-masked Gr membranes recorded in LiCl, $CaCl_2$, and $MgCl_2$ solution.

Supplementary Tables

Supplementary Table 1 Selected physical and thermodynamic characteristics of the tested ions[19-22].

Ions	Crystal radius (Å)	Hydrated radius (Å)	Coordination number	Hydration free energy (kJ mol^{-1})
K^+	1.38	3.31	6.6	321
Na^+	1.02	3.58	5.9	405
Li^+	0.76	3.82	4.5	515
Ca^{2+}	1.00	4.12	8.0	1592
Mg^{2+}	0.72	4.28	6.0	1922

Supplementary Table 2 Comparison of the membranes for Li^+/Mg^{2+} separation in concentration-driven single-ion sieving tests.

**Membranes**	**Li^+ normalized permeation rate (m/h)**	**Li^+/Mg^{2+} selectivity**	**Feed solution concentration (mol/L)**	**Reference**
Nafion 117	1.15×10^{-3}	2.07	0.01	[9]
Nafion 212	7.76×10^{-4}	1.23	1	[23]
PIM	1.27×10^{-3}	32.60	1	[23]
PIM	6×10^{-5}	14.60	1	[23]
PIM	6.10×10^{-4}	16.10	1	[23]
PIM	6.63×10^{-3}	7.53	1	[23]
PIM	1.66×10^{-3}	2.10	1	[23]
Mxene	7×10^{-3}	8.75	0.2	[24]
Mxene	$\sim7\times10^{-5}$	2.33	0.5	[25]
Mxene	$\sim2.10\times10^{-5}$	3.33	0.5	[25]
Mxene	1.72×10^{-3}	1.22	0.2	[26]
Mxene	7×10^{-5}	2.60	0.2	[26]
COF	$\sim3.40\times10^{-4}$	64	0.1	[27]
COF	5.53×10^{-4}	217	0.1	[28]
i-CMP	3.26×10^{-3}	17.16	0.01	[9]
CMP	3.99×10^{-3}	4.84	0.01	[9]
CMP	8.04×10^{-3}	1.28	0.01	[8]
CMP	4.12×10^{-3}	1.39	0.1	This work
CMP	1.05×10^{-3}	1.90	0.1	This work
CMP	4.88×10^{-5}	1.24	0.1	This work
Porous Graphene	5.11×10^{-4}	25.08	0.1	This work
Porous Graphene	9.61×10^{-4}	12.99	0.1	This work
Porous Graphene	1×10^{-3}	18.53	0.1	This work
Porous Graphene	2.31×10^{-4}	55.79	0.1	This work
CMP-masked graphene	1.99×10^{-4}	313.39	0.1	This work
CMP-masked graphene	1.03×10^{-4}	232.30	0.1	This work
CMP-masked graphene	1.05×10^{-4}	236.83	0.1	This work
CMP-masked graphene	2.58×10^{-4}	207.60	binary ions	This work

Supplementary Table 3 Comparison of the membranes for K^+/Mg^{2+} separation in concentration-driven single-ion sieving tests.

**Membranes**	**K^+ normalized permeation rate (m/h)**	**K^+/Mg^{2+} selectivity**	**Feed solution concentration (mol/L)**	**Reference**
Nafion 212	1.53×10^{-3}	2.44	1	[23]
i-CMP	7.67×10^{-2}	40.90	0.01	[9]
CMP	1.01×10^{-2}	7.07	0.01	[9]
CMP	1.05×10^{-2}	1.67	0.01	[8]
PA	$\sim6\times10^{-5}$	2	0.1	[5]
PIM	1.08×10^{-2}	12.30	1	[23]
PIM	1.22×10^{-3}	31.30	1	[23]
PIM	2×10^{-4}	48.80	1	[23]
PIM	1.60×10^{-3}	42.10	1	[23]
PIM	2.40×10^{-3}	3.01	1	[23]
Mxene	4.70×10^{-3}	5.88	0.2	[24]
Mxene	$\sim4.4\times10^{-4}$	4.40	0.5	[29]
Mxene	$\sim1.4\times10^{-4}$	2.80	0.5	[29]
Mxene	1.89×10^{-3}	1.39	0.2	[26]
Mxene	1.27×10^{-4}	4.62	0.2	[26]
GO	5.50×10^{-4}	9.70	0.1	[30]
GO	1.90×10^{-5}	50	0.1	[30]
GO	3.60×10^{-5}	11	0.1	[30]
GO	8.9×10^{-6}	90.30	0.1	[30]
GO	9.90×10^{-4}	3.69	0.5	[22]
GO	2.72×10^{-3}	9.11	0.5	[22]
COF	1.95×10^{-3}	764.71	0.1	[28]
CMP-masked graphene	2.93×10^{-4}	530.39	0.1	This work

Supplementary References

1 Zhou, Z., Hu, Y., Wang, Q. & Mi, B. Carbon nanotube-supported polyamide membrane with minimized internal concentration polarization for both aqueous and organic solvent forward osmosis process. *Journal of Membrane Science* **611**, 118273 (2020).

2 Zhu, Y. *et al.* Single-walled carbon nanotube film supported nanofiltration membrane with a nearly 10 nm thick polyamide selective layer for high-flux and high-rejection desalination. *Small* **12**, 5034-5041 (2016).

3 Zhou, Z. *et al.* Electropolymerization of robust conjugated microporous polymer membranes for rapid solvent transport and narrow molecular sieving. *Nature Communications* **11**, 5323 (2020).

4 Bepete, G. *et al.* Surfactant-free single-layer graphene in water. *Nature Chemistry* **9**, 347-352 (2017).

5 Gao, S. *et al.* Ultrathin polyamide nanofiltration membrane fabricated on brush-painted single-walled carbon nanotube network support for ion sieving. *ACS nano* **13**, 5278-5290 (2019).

6 Hauser, D., Septiadi, D., Turner, J., Petri-Fink, A. & Rothen-Rutishauser, B. From bioinspired glue to medicine: polydopamine as a biomedical material. *Materials* **13**, 1730 (2020).

7 Wu, Y. & Aluru, N. Graphitic carbon–water nonbonded interaction parameters. *The Journal of Physical Chemistry B* **117**, 8802-8813 (2013).

8 Zhou, Z. *et al.* Precise sub-angstrom ion separation using conjugated microporous polymer membranes. *Acs Nano* **15**, 11970-11980 (2021).

9 Zhou, Z. *et al.* Flexible ionic conjugated microporous polymer membranes for fast and selective ion transport. *Advanced Functional Materials* **32**, 2108672 (2022).

10 Suárez-García, S., Sedó, J., Saiz-Poseu, J. & Ruiz-Molina, D. Copolymerization of a catechol and a diamine as a versatile polydopamine-like platform for surface functionalization: The case of a hydrophobic coating. *Biomimetics* **2**, 22 (2017).

11 Batul, R., Bhave, M., J. Mahon, P. & Yu, A. Polydopamine nanosphere with in-situ loaded gentamicin and its antimicrobial activity. *Molecules* **25**, 2090 (2020).

12 Zangmeister, R. A., Morris, T. A. & Tarlov, M. J. Characterization of polydopamine thin films deposited at short times by autoxidation of dopamine. *Langmuir* **29**, 8619-8628 (2013).

13 Li, J. *et al.* Fabrication and characterization of carbon nanotubes-based porous composite forward osmosis membrane: Flux performance, separation mechanism, and potential application. *Journal of Membrane Science* **604**, 118050 (2020).

14 Deng, L., Wang, Q., An, X., Li, Z. & Hu, Y. Towards enhanced antifouling and flux performances of thin-film composite forward osmosis membrane via constructing a sandwich-like carbon nanotubes-coated support. *Desalination* **479**, 114311 (2020).

15 He, G. *et al.* High-permeance polymer-functionalized single-layer graphene membranes that surpass the postcombustion carbon capture target. *Energy & Environmental Science* **12**, 3305-3312 (2019).

16 Zhou, J. *et al.* Highly conductive and vanadium sieving Microporous Tröger's Base Membranes for vanadium redox flow battery. *Journal of Membrane Science* **620**, 118832 (2021).

17 Zhang, H. *et al.* Ultrafast selective transport of alkali metal ions in metal organic frameworks with subnanometer pores. *Science advances* **4**, eaaq0066 (2018).

18 Li, X. *et al.* Fast and selective fluoride ion conduction in sub-1-nanometer metal-organic framework channels. *Nature communications* **10**, 2490 (2019).

19 Zhu, H., Wang, Y., Fan, Y., Xu, J. & Yang, C. Structure and transport properties of water and hydrated ions in nano-confined channels. *Advanced Theory and Simulations* **2**,

1900016 (2019).
20 Nightingale Jr, E. Phenomenological theory of ion solvation. Effective radii of hydrated ions. *The Journal of Physical Chemistry* **63**, 1381-1387 (1959).
21 Marcus, Y. A simple empirical model describing the thermodynamics of hydration of ions of widely varying charges, sizes, and shapes. *Biophysical chemistry* **51**, 111-127 (1994).
22 Zhang, M. *et al.* Designing biomimic two-dimensional ionic transport channels for efficient ion sieving. *ACS nano* **15**, 5209-5220 (2021).
23 Tan, R. *et al.* Hydrophilic microporous membranes for selective ion separation and flow-battery energy storage. *Nature Materials* **19**, 195-202 (2020).
24 Ren, C. E. *et al.* Charge-and size-selective ion sieving through Ti3C2T x MXene membranes. *The journal of physical chemistry letters* **6**, 4026-4031 (2015).
25 Zhu, J. *et al.* Precisely tunable ion sieving with an Al13–Ti3C2T x lamellar membrane by controlling interlayer spacing. *ACS nano* **14**, 15306-15316 (2020).
26 Kang, Y. *et al.* Nanoconfinement enabled non-covalently decorated MXene membranes for ion-sieving. *Nature Communications* **14**, 4075 (2023).
27 Bing, S. *et al.* Bio-inspired construction of ion conductive pathway in covalent organic framework membranes for efficient lithium extraction. *Matter* **4**, 2027-2038 (2021).
28 Sheng, F. *et al.* Efficient Ion Sieving in Covalent Organic Framework Membranes with Sub-2-Nanometer Channels. *Advanced Materials* **33**, 2104404 (2021).
29 Wang, J. *et al.* Ion sieving by a two-dimensional Ti3C2T x alginate lamellar membrane with stable interlayer spacing. *Nature communications* **11**, 3540 (2020).
30 Qian, Y. *et al.* Enhanced ion sieving of graphene oxide membranes via surface amine functionalization. *Journal of the American Chemical Society* **143**, 5080-5090 (2021).
